# Measure selection for an electronic patient-reported outcome (ePRO) system for CAR T-cell therapy patients: a modified Delphi consensus study

**DOI:** 10.1016/j.eclinm.2025.103256

**Published:** 2025-05-28

**Authors:** Sarah E. Hughes, Foram Khatsuria, Christel McMullan, Karen L. Shaw, Anita Walker, Francesca Kinsella, David Burns, Olalekan L. Aiyegbusi, Elin Haf Davies, John Ansell, Evelyn Chakera, Charles Craddock, Alastair Denniston, Rebecca Lloyd, Paul Ferguson, Ronjon Chakraverty, Melanie Calvert

**Affiliations:** a Centre for Patient Reported Outcome Research, University of Birmingham, Birmingham, UK; b National Institute of Health and Care Research (NIHR) Blood and Transplant Research Unit (BTRU) in Precision Cellular Therapeutics, University of Birmingham, Birmingham, UK; c NIHR Biomedical Research Centre, University of Birmingham, Birmingham, UK; d NIHR Applied Research Collaboration (ARC) West Midlands, University of Birmingham, Birmingham, UK; e University Hospitals Birmingham NHS Foundation Trust, Birmingham, UK; f Aparito Limited, Wrexham, UK; g BTRU Patient and Public Involvement and Engagement (PPIE) Group, Birmingham, UK; h Birmingham Health Partners Centre for Regulatory Science and Innovation, Birmingham, UK; i NIHR-Supported Incubator in AI & Digital Healthcare, Birmingham, UK; j Radcliffe Department of Medicine, University of Oxford, Oxford, UK

**Keywords:** Delphi, Chimeric antigen receptor T-cell therapy, Patient-reported outcome, ePRO, Digital health

## Abstract

**Background:**

Chimeric Antigen Receptor (CAR) T-cell therapies are effective for treating haematological cancers but carry risks of toxicity and substantial symptom burden. Patient-reported outcomes (PROs) could significantly enhance clinical management for patients undergoing these treatments. However, guidance on selection of PRO measures for monitoring adverse event and quality of life after CAR T-cell therapy is limited. This study aimed to achieve consensus among patients and healthcare professionals on the selection of PRO measures for an electronic PRO (ePRO) system for CAR T-cell therapy clinical settings.

**Methods:**

Two-round modified Delphi study (online survey and consensus meeting) conducted from December 2023 to January 2024 to select PRO measures for the ePRO system, guided by a conceptual framework with four measurement domains: symptom burden, impacts of cancer and CAR T-cell therapy, treatment tolerability, and health-related quality of life (HRQoL). Database searches (PubMed, ePROVIDE, COSMIN, and COMET) and licensing websites of cancer-specific PRO measures identified 113 PRO measures. Measures were pre-specified for treatment tolerability and HRQoL domains and concept mapping established conceptual coverage for the remaining domains. Seven PRO measures were shortlisted and prespecified inclusion thresholds and stopping criteria guided Delphi panel selection. Registration: ISRCTN11232653.

**Findings:**

Nineteen participants (5 CAR T-cell patients, 14 healthcare professionals/researchers) recruited from a UK National Health Service (NHS) cellular therapy centre and professional networks took part in Round One (Delphi online survey). Shortlisted measures were rated for relevance, comprehensiveness, and ease of understanding for the symptom burden and impacts of cancer and CAR T-cell treatment domains. Consensus was achieved after Round One, precluding the requirement for Round 2 (consensus meeting). The Symptom Burden Questionnaire™ (SBQ™) and the Quality of Life in Adult Cancer Survivors (QLACS) were selected to represent the Symptom Burden and Impacts domains, respectively. These measures, EQ5D-5L, measuring HRQoL, and Functional Assessment of Chronic Illness Therapy–Item GP5 (FACT-GP5), single-item global indicator of cancer treatment tolerability, will be included in the ePRO system.

**Interpretation:**

In the absence of guidance on PRO measure selection for CAR T-cell therapies, consensus-based methods represent an important step towards use of PROs with this clinical population. Modest sample size and representativeness of the patient subgroup are limitations of this study.

**Funding:**

This study is funded by the 10.13039/501100000272National Institute for Health and Care Research (NIHR) Blood and Transplant Research Unit in Precision Cellular Therapeutics (NIHR203339). The views expressed are those of the authors and not necessarily those of the NIHR, NHS Blood and Transplant, or the Department of Health and Social Care.

**Research in context**

**Evidence before this study**

Patient-reported outcomes (PROs), self-report measures of how a patient feels and functions, have been identified as potentially valuable for the clinical management of patients who are recipients of CAR T-cell therapies. However, there are inconsistencies in the evidence on PRO measure selection for this patient group. We conducted a rapid review and Delphi consensus study with patients and healthcare professionals to identify and select PRO measures to include in an electronic PRO (ePRO) system for use in clinical practice.

**Added value of this study**

This is the first study to select PRO measures for CAR T-cell therapies using consensus methods and represents an important step in the development of co-designed tools to support clinical management of this growing patient group. This study adds value as current guidelines and core outcome sets do not provide specific recommendations on the selection of PRO measures for use with this clinical population. Measures were selected by a Delphi consensus process to collect CAR T-cell therapy recipients’ perspectives on symptom burden, wider impacts of CAR T-cell therapy and cancer treatments, global treatment tolerability, and health-related quality of life.

**Implications of all the available evidence**

Findings from this Delphi study provide guidance on the selection of currently available PRO measures for digital implementation as part of routine care for CAR T-cell therapy patients with haematological cancers. As more CAR-T-specific PRO measures become available, updates to these recommendations will be required. ePRO systems for CAR T-cell therapy could support remote monitoring of patients for acute, late, and long-term effects of CAR T-cell therapy with the benefit of enhancing clinical care and improving patient experience.

## Introduction

Chimeric antigen receptor (CAR) T-cell therapy is an adoptive cell therapy in which the T-cell is modified to target and eradicate cancer cells more effectively. CAR T-cell therapies have been approved for patients with specific haematological malignancies with candidacy expected to expand rapidly to include other cancers in future.

CAR T-cell therapies have the potential to induce long-lasting remission; however, treatment is associated with severe, acute toxicities such as cytokine release syndrome (CRS) and neurological toxicities (e.g., immune effector cell–associated neurologic toxicity syndrome, ICANS) that can be fatal.^1^^,^^2^ The unique toxicity profile of CAR T-cell therapies necessitates close monitoring of patients immediately following infusion, requiring patients to endure lengthy hospitalisation and/or move to accommodation in close proximity to their CAR T-cell therapy centre for close surveillance by their clinical team. Growing numbers of long-term survivors means there is increasing recognition of the need to manage long-term effects of CAR T-cell therapy including late-onset or persistent neurotoxicity, cytopenia, infection, secondary malignancies, organ dysfunction, fertility considerations, fatigue, and psychosocial concerns.^3^^,^^4^

Patient-reported outcomes (PROs) are direct reports from patients about their health, collected without interpretation by a clinician or anyone else.^5^ PROs are well-established in the field of oncology and remote monitoring using electronic PROs (ePROs) has been shown to reduce emergency attendance and hospital admissions, improve survival, and improve the quality of life in cancer patients with solid organ tumours receiving chemotherapy.^6^^,^^7^ There are a growing number of studies to suggest CAR T-cell therapies impact PROs, largely as a result of central nervous system (CNS) effects.^8^ As such, PROs hold potential for monitoring the immediate and long-term side effects of CAR T-cell therapy, as well as other dimensions of importance to patients, such health-related quality of life (HRQoL).^7^^,^^8^ Remote ePRO monitoring could provide an effective and affordable means to support treatment decision-making through the delivery of real-time communication about how a patient is feeling, a key consideration for the development of patient-centred care pathways where patients often transition from specialist CAR-T centres back to local haemato-oncology services for continued care. Patient monitoring using PROs is particularly relevant given the current interest in establishing outpatient CAR T-cell therapy as standard of care and in the UK.^9^

Whilst the number of studies of CAR T-cell therapies reporting PROs is growing, the number of studies describing the implementation of PROs in the context of routine clinical care of CAR-T patients remains limited.^10^ For PROs to be utilised meaningfully in the context of the clinical management of CAR T-cell therapy patients, consideration needs to be given to how best to capture these data. Best practice guidance in both Europe and in the United States advocates for electronic capture of PROs (ePROs) for symptom monitoring. Key to implementation is the selection of appropriate PRO measures with stakeholder involvement.^11^ The PRO-CAR-T Study is a mixed-methods, multi-phase study that aims to develop and evaluate the feasibility of a new ePRO system for use in the clinical care of patients receiving CAR T-cell therapies.^12^ Here we report on a consensus building process to select PRO measures for inclusion in the PRO-CAR-T ePRO system.

## Methods

### Aims and objectives

**Fig. 1:**
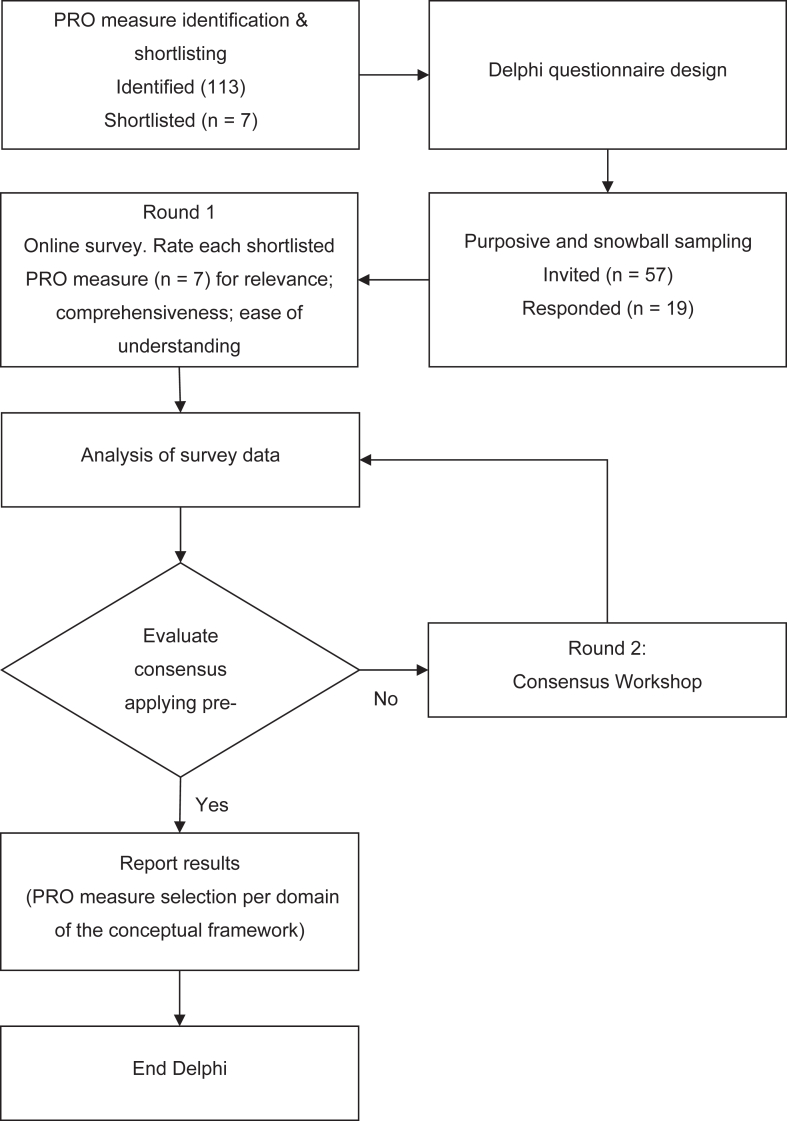
Study flow diagram showing the process of PRO measure identification and consensus-based selection.

This modified two-round Delphi consensus study aimed to select PRO measures to include in an electronic patient-reported outcome (ePRO) data capture system for monitoring of patients with haematological malignancies following CAR T-cell therapy. Our study builds on previous work that developed a conceptual framework for the new ePRO system.^13^ The process of PRO measure shortlisting and consensus-based selection is shown in Fig. 1.

### Ethics

A favourable ethical opinion was granted by the Health Research Authority, Health and Social Care Research Ethics Committee B (Ref: 23/NI/0104) and the study was prospectively registered (Ref: ISRCTN11232653 https://doi.org/10.1186/ISRCTN11232653). Surveys were completed by participants anonymously and all data were stored securely at the University of Birmingham with only authorised members of the study team at the University of Birmingham having access. Informed consent was obtained from all Delphi participants. In Round 1 participants gave electronic consent before completing the online Delphi survey.

### ePRO conceptual framework

A conceptual framework, as a structured representation of the concepts and domains intended to be measured, summarises relevant experience of patients in the target population and the types of clinical outcome assessments for each concept.^14^ The ePRO conceptual framework was developed from a rapid literature review with confirmation of conceptual relevance and representativeness by clinicians, patients, and their family members.^13^ The framework grouped 109 concepts of importance into four conceptual domains: symptom burden (95 constructs, 15 subdomains broadly representing different body systems), impacts of disease and treatment (12 constructs, 4 subdomains), tolerability, and quality of life (see Supplementary file, Appendix 1).

### PRO measure identification and shortlisting

Candidate PRO measures were identified from multiple sources: 1) a rapid review of the literature on PROs in CAR T-cell therapy, the methods of which are reported elsewhere,^13^ and searches of; 2) COSMIN database of systematic reviews of PRO instruments (https://www.cosmin.nl/); 3) ePROVIDE PROQOLID database (https://eprovide.mapi-trust.org/); 4) and three PRO measure websites (FACIT (https://www.facit.org), MD Anderson (https://www.mdanderson.org), and PROMIS (https://www.promishealth.org). The EORTC (https://qol.eortc.org/item-library/) and Patient-Reported Outcomes version of the Common Terminology Criteria for Adverse Events (PRO-CTCAE) (https://healthcaredelivery.cancer.gov/pro-ctcae/instrument-pro.html) item libraries were included in the long list of PRO measures identified in the rapid literature review and therefore were not included in the database and website searches. Searches were conducted separately for each domain of the framework using tailored search terms (see Supplementary file, Appendix 2) during the period November 2, 2023 to December 8, 2023. To arrive at a shortlist of PRO measures for the Delphi survey, items from the candidate measures were charted against the Symptom Burden and Impact domains to establish conceptual coverage per domain. Coverage was reported using descriptive statistics (see Supplementary file, Appendix 3). Measures for Quality of Life and Tolerability domains were pre-specified by the study team and included the EQ5D-5L as a measure of health-related quality of life (HRQoL) and the single item Functional Assessment of Chronic Illness Therapy–Item GP5 (FACT-GP5) (i.e., “I am bothered by side effects of treatment”) as a measure of treatment tolerability. The FACT-GP5 was selected as a summary measure of treatment-related tolerability to supplement the reporting of specific symptoms and side effects captured by the Symptom Burden domain of the conceptual framework. An understanding of a patient's overall tolerability of treatment is crucial when managing complex treatments like CAR T-cell therapy with its potential for severe side effects. The FACT-GP5 is validated in cancer populations, used routinely for evaluating self-reported treatment tolerability in oncology trials, is not burdensome to complete, and has been shown to be meaningful to people with cancer.^15^ The EQ-5D-5L is a well-established patient-reported outcome (PRO) measure of health-related quality of life with evidence of use with CAR T-cell recipients and in trials of advanced therapy medicinal products (ATMPs) and is recommended for local health reimbursement in the UK.^16^, ^17^, ^18^

A PRO measure was included if it met pre-determined eligibility criteria. Measures were shortlisted if they had at least one item mapped per subdomain and met one of the following criteria: 1) provided ≥70% coverage of the 15 Symptom Burden subdomains, or 2) provided coverage of all four Impacts of Disease and Treatment subdomains. A PRO measure was included if it was published in English, had a recall period of seven days or less, was suitable for electronic delivery, and was specified as suitable for clinical use. Given the paucity of PRO measures with evidence of validation in the CAR-T patient population, generic measures and cancer-specific measures validated in related but different cancer patient populations (e.g., bone marrow transplantation) were considered for inclusion if they met the threshold for conceptual coverage (see Supplementary file, Appendix 4).

Candidate PRO measures were screened for evidence of their measurement properties, applying a modified COSMIN checklist developed for a previous Delphi study of PRO measure selection.^19^ Reports of measure development and content validity studies were located, and two team members (SH, FK) independently assessed the shortlisted measures to ascertain the availability of evidence of the measures’ psychometric measurement properties (see Supplementary file, Appendix 5). Disagreements were resolved through discussion and involved a third reviewer (MC) where necessary to reach consensus. Evidence was collated and presented in table format to Delphi participants in the online survey (see Supplementary file, Appendix 6).

### Delphi consensus process

A two-round modified Delphi consensus process was conducted over a one-month period (December 20, 2023 to January 19, 2024). CAR T-cell therapy patients and their family members, healthcare professionals, academics, policymakers and representatives from third sector organisations were invited to take part. The Delphi method is a consensus-based approach routinely used in outcome measure selection and Core Outcome Set development.^20^ Clinical staff at a single, regional National Health Service (NHS) CAR T-cell therapy centre screened the CAR-T patient caseload, sending invitations to all patients who met the study eligibility criteria (i.e., >18+ years, able to undertake the protocol activities, and able to provide their informed consent). Patients were also recruited via the National Institute for Health and Care Research (NIHR) Blood and Transplant Research Unit (BTRU) Patient and Public Involvement and Engagement (PPIE) group and an advertisement posted on social media (i.e., closed CAR-T patient support group page). Family members of patient participants were invited to take part through snowball sampling. Healthcare professionals from six NHS CAR T-cell therapy regional centres and known contacts, including researchers, policymakers and regulators, linked to the NIHR Blood and Transplant Research Unit in Precision Cellular Therapeutics were also invited to take part.

Round 1 comprised an online survey administered using the university-approved web-based platform Smart Survey (www.smartsurvey.co.uk). Responses of individual participants were anonymised to protect individual participant confidentiality and participants were required to give their informed consent in order to access and complete the survey. Participants were provided with the shortlist of PRO measures (and corresponding instrument card) mapped to the symptom burden and impacts of disease and treatment domains respectively. For each domain, participants reviewed and rated each of the shortlisted PRO measures against three criteria: 1) relevance (the degree to which all the items within a PRO measure are relevant for the construct of interest within a specific population and context of use); 2) comprehensiveness (whether all key concepts are included in the PRO measure); and 3) ease of understanding as criteria for good content validity as recommended by COSMIN.^21^ Participants responded using either a 6-point or 4-point ordinal scales with values ranging from 0 = Not at all relevant/comprehensive to 5 = Extremely relevant/comprehensive or 0 = Very difficult to understand to 3 = Very easy to understand. Free text boxes were available to capture additional feedback (see Supplementary file, Appendix 7 for example screenshot from the online survey).

### Statistics

Participants’ ratings of relevance and comprehensiveness were converted from a 6-point scale to dichotomous scores such that score 0 to 2 returned a “no” response and scores from 3 to 6 were assigned a “yes” response. For ease of understanding, a 4-point scale was used. A “no” response was assigned to scores of 0 or 1 and a “yes” response was assigned to scores of 2 or 3. Scores were reported using descriptive statistics (frequency counts and percentages) in Excel. For each criterion, an indicator of agreement was applied when >70% of scores = “yes” (i.e., a score of 3–6). An indicator of “some doubt” (?) was applied when 50%–70% of participants responded “yes” and an indicator of “substantial doubt” (??) was applied when less than 50% of participants responded “yes” to an indicator. Consistency of scoring across participant subgroups, as an indicator of both response stability and content validity, was explored for each criterion. Fisher's exact test, due to the small sample size, was used to determine if there was a significant association between stakeholder group and voting for each criterion of relevance, comprehensiveness, and ease of understanding per domain for each PROM. A p-value greater than 0.05 indicated no statistically significant difference in voting, providing evidence of consistency and uniformity across participant subgroups.

There is no definitive guidance on thresholds for consensus/agreement in Delphi studies, with values ranging typically between 70% and 80%.^20^^,^^22^^,^^23^ We adopted, *a priori,* criteria used by Murphy et al. but applied a more stringent 70% threshold as an indicator of agreement, in line with previously published Delphi studies.^20^^,^^23^, ^24^, ^25^ A measure was accepted to go forward for Round 2 if the prespecified indicators of agreement (✓) were returned for all three criteria of relevance, comprehensiveness, and ease of understanding (✓✓✓). Consensus was defined as agreement for all three criteria for a single PRO measure per domain. If consensus was reached, it was accepted as the PRO measure for that domain. If no PRO measures achieved three agreement indicators, then all PRO measures with a maximum of one doubt indicator (e.g., ?✓✓ or ✓??✓) were accepted to go forward for discussion and voting in Round 2. If multiple PRO measures within a domain had indicators of agreement for all three criteria (✓✓✓), these PRO measures were sent forward for discussion in Round 2. PRO measures receiving two or more doubt (e.g., ??✓ or ??✓??) indicators were excluded. In Round 2, iterative rounds of discussion and voting were scheduled until consensus was achieved for a single PRO measure for each domain. The same criterion indicators for consensus applied in Round 2 as those used in Round 1. For the overall study, the criterion to stop the Delphi process was defined as consensus agreement for a single PRO measure for each domain, with evidence of consistency in responses across participant subgroups (defined as patients and healthcare professionals/researchers) within a round.

### Role of the funding source

The funder had no role in study design; in the collection, analysis, and interpretation of data; in the writing of the report; and in the decision to submit the paper for publication.

## Results

### PRO measure identification and shortlisting

**Fig. 2:**
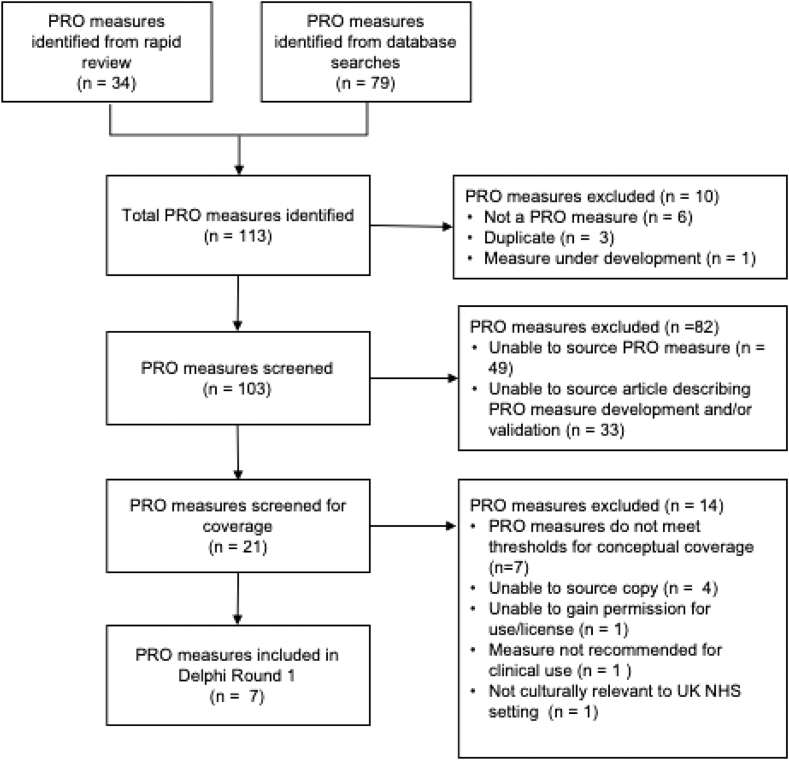
Overview of the PRO measure shortlisting process.

The rapid review and database searches identified 113 individual PRO measures for consideration. After removal of duplicates (n = 3), measures that were not patient-reported (n = 6), and measures under development (n = 1), 103 instruments were sent forward for screening. Of these, 82 instruments were excluded for failing to meet the pre-specified thresholds for subdomain coverage, defined as either complete coverage (100%) of the Impacts subdomains (4/4) or >70% coverage of the Symptom Burden subdomains (11/15). Coverage was determined as having at least one item mapped to a given subdomain. The remaining 14 PRO measures were appraised for recall period, licensing requirements, and suitability for clinical use. Seven PRO measures were excluded for one of the following reasons: 1) a copy of the questionnaire form could not be sourced (n = 4), unable to gain permission/license from developer (n = 1), 2) PRO measure was not recommended for clinical practice (n = 1), or 3) the measure was not culturally relevant for the UK (n = 1). Fig. 2 shows a flow diagram of the PRO measure shortlisting process.

Overall, seven PRO measures met the eligibility criteria for selection and were included for consideration by the Delphi consensus panel. These measures were the Functional Assessment of Cancer Therapy–Bone Marrow Transplant (FACT-BMT), MD Anderson Symptom Inventory CAR module (MDASI-CAR), Quality of Life in Bone Marrow Transplant Survivors (QoL-BMT), Symptom Burden Questionnaire (SBQ), Myeloma Patient Outcome Scale (MyPOS), Supportive Care Needs Survey—Short Form 34 (SCNS-SF34), and the cancer subscales of the Quality of Life in Adult Cancer Survivors (QLACS).^26^, ^27^, ^28^, ^29^, ^30^, ^31^, ^32^ Two measures, FACT-BMT and QoL-BMT, met the threshold for inclusion for both domains. Tables 1 and 2 present the domains and sub-domains of the conceptual framework covered by each of the PRO measures shortlisted for review by the Delphi participants.

**Table 1:** Conceptual mapping of the shortlisted PRO measures to the symptom Burden domain of the ePRO conceptual framework.

Sub-domain	FACT (BMT)	MDASI-CAR	QoL-BMT	SBQ
Neurological symptoms (n = 17)	2	6	2	8
Skin and hair-related symptoms (n = 8)	0	0	0	4
Ear, nose and throat (n = 10)	1	1	3	5
Clinical signs (n = 9)	1	4	1	6
Cardiopulmonary (n = 4)	1	1	1	1
Psychological (n = 8)	1	2	4	3
Gastrointestinal (n = 6)	1	3	2	6
Fever-related symptoms (n = 3)	0	1	0	3
Infection (n = 5)	1	0	0	1
Sleep (n = 5)	2	2	1	3
Musculoskeletal (n = 4)	0	0	0	3
Sexual functioning (n = 3)	1	1	2	2
Fatigue (n = 3)	1	1	1	1
Vision/eye-related symptoms (n = 3)	1	0	1	2
Interference (n = 7)	5	6	6	6
Total symptom concepts (N = 95)	18	28	24	54
Sub-domains represented (15/15) (%)	12 (80%)	11 (73%)	11 (73%)	15 (100%)

FACT (BMT), Functional Assessment of Cancer Therapy–Bone Marrow Transplant; MDASI-CAR, MD Anderson Symptom Inventory CAR module; QoL-BMT, Quality of Life Bone Marrow Transplant Survivors; SBQ Symptom Burden Questionnaire.

**Table 2:** Conceptual mapping of the shortlisted PRO measures to the Impacts of Disease and Treatment domain of the ePRO conceptual framework.

Sub-domain	FACT (BMT)	QoL-BMT	SCNS-SF	MY-POS	QLACS
Psychosocial Impacts (n = 4)	4	4	2	1	4
Perception of Risk (n = 2)	2	1	1	2	1
Financial Impacts (n = 1)	1	1	1	1	1
Support Requirements (n = 4)	2	2	3	3	1
Total concepts (N = 12)	9	8	7	7	7
Total sub-domain coverage (4/4) (%)	4 (100%)	4 (100%)	4 (100%)	4 (100%)	4 (100%)

FACT (BMT), Functional Assessment of Cancer Therapy–Bone Marrow Transplant; QoL-BMT, Quality of Life Bone Marrow Transplant Survivors; SCNS-SF34, Supportive Care Needs Survey—Short Form 34; MYPOS, Myeloma Patient Outcome Scale; QLACS, Quality of Life in Adult Cancer Survivors.

### Delphi consensus process

Fifty-seven participants were invited to take part in Round 1 of the Delphi process. Complete responses were received from 19 participants, a response rate of 33%. Group membership included CAR T-cell therapy patients (adults aged 18+ years, n = 5), clinicians with expertise in CAR T-cell therapies (n = 10), academics/researchers (n = 3), and clinical scientists (n = 1).^33^ Eleven (58%) of participants were female and 16 (84%) were of White ethnicity (Table 3).

**Table 3:** Participants’ demographic characteristics for the Delphi survey (N = 19).

	N (%)
Participant group	
Academic	3 (16%)
Clinician scientist^a^	1 (5%)
Family member/caregiver	0 (0%)
Healthcare professional	10 (53%)
Patient	5 (26%)
Sex	
Female	11 (58%)
Male	8 (42%)
Age	
18–29 years	2 (10.5%)
30–39 years	1 (5%)
40–49 years	7 (37%)
50–59 years	3 (16%)
60–69 years	4 (21%)
70+ years	2 (11%)
Ethnicity^b^	
Asian/Asian British	2 (11%)
Black/African/Caribbean/Black British	0 (0%)
Mixed/Multiple Ethnic Group	1 (5%)
White	16 (84%)
Other ethnic group	0 (0%)

a A clinician scientist is a healthcare professional who combines research with clinical practice.^33^

b Ethnic group classifications: https://www.ons.gov.uk/census/census2021dictionary/variablesbytopic/ethnicgroupnationalidentitylanguageandreligionvariablescensus2021/ethnicgroup/classifications.

### Missing data

Data were not missing due to the requirement for participants to complete all data fields to submit the survey.

### Analysis

Across both domains, participant responses indicated they were generally in favour of the shortlisted measures, with percentage of “yes” votes ranging from 37% to 84% for the three inclusion criteria (Table 4). For the symptom burden domain, one PRO measure (Symptom Burden Questionnaire™) exceeded the minimum 70% threshold for acceptance for all three criteria (i.e., ✓✓✓), indicating a consensus on PRO measure selection for this domain. No measures returned a single indicator of either “some” or “substantial” doubt. Three measures had two or more indicators of doubt and were therefore excluded (i.e., FACT-BMT, MDASI-CAR, QoL-BMT). For the “Impacts of Disease and Treatment” domain, QLACS was the only measure to reach agreement on all three indicators (e.g., ✓✓✓), indicating consensus agreement. Two measures (i.e., FACT (BMT) and QoL-BMT) had one indicator each of either “some” (e.g.,?) or “substantial” doubt (e.g., ??) and two measures were excluded on the basis of having two or more indicators of “some” or “substantial” doubt (i.e., MyPOS and SCNC-SF34). Table 5 summarises the indicators of agreement and results of the Delphi survey (Round 1).

**Table 4:** Percentage of respondents voting “yes” for each of the consensus indicators.

Domain	PRO measure	Percentage of respondents voting “yes”		
		Relevance	Comprehensiveness	Ease of understanding
Symptom Burden	FACT (BMT)	58%	53%	79%
	MDASI-CAR	68%	63%	68%
	QoL-BMT	63%	63%	47%
	SBQ	79%	79%	74%
Impacts of Disease and Treatment	FACT (BMT)	63%	74%	84%
	MyPOS	37%	53%	84%
	QoL-BMT	74%	74%	58%
	SCNC-SF34	63%	68%	68%
	QLACS	74%	74%	84%

FACT (BMT), Functional Assessment of Cancer Therapy–Bone Marrow Transplant; MDASI-CAR, MD Anderson Symptom Inventory CAR module; QoL-BMT, Quality of Life Bone Marrow Transplant; SBQ, Symptom Burden Questionnaire; MYPOS, Myeloma Patient Outcome Scale; SCNS-SF34, Supportive Care Needs Survey—Short Form 34; QLACS, Quality of Life in Adult Cancer Survivors.

**Table 5:** Indicators of agreement and results of the Delphi online survey.

Domain	PRO measure	Round 1			Round 1 decision
		R	C	E	
Symptom Burden	FACT (BMT)	?	?	✓	Exclude
	MDSAI-CAR	?	?	?	Exclude
	QoL-BMT	?	?	??	Exclude
	SBQ	✓	✓	✓	Include
Impacts of Disease and Treatment	FACT (BMT)	?	✓	✓	Round 2^a^
	MY-POS	??	?	✓	Exclude
	QoL-BMT	✓	✓	?	Round 2^a^
	QLACS	✓	✓	✓	Include
	SCNS-SF34	?	?	?	Exclude

R, relevance; C, comprehensiveness; E, Ease of understanding; ✓, agreement (>70% responded “yes”; ?, some doubt (50%–70% responded “yes”); ??, substantial doubt (<50% responded “yes”); FACT(BMT), Functional Assessment of Cancer Therapy–Bone Marrow Transplant; MDASI-CAR, MD Anderson Symptom Inventory CAR module; QoL-BMT, Quality of Life Bone Marrow Transplant Survivors; SBQ, Symptom Burden Questionnaire; MYPOS, Myeloma Patient Outcome Scale; QLACS, Quality of Life in Adult Cancer Survivors; SCNS-SF34, Supportive Care Needs Survey—Short Form 34.

a Proceed to Round 2 if no measures within a domain receive agreement indicators for all three outcomes.

The percentage of participants giving a “yes” response ranged from 58% to 69% for CAR T-cell therapy patients and from 66% to 74% for healthcare professionals/researchers (Table 6).

**Table 6:** Percentage of participants per subgroup giving a “yes” response (score of ≥ 3 for relevance or comprehensiveness or ≥ 2 for ease of understanding).

	CAR T-cell therapy patients (%)	Healthcare professionals/researchers (%)
Relevance	58	67
Comprehensiveness	69	66
Ease of Understanding	67	74

Fisher's Exact Test was used to examine the association between stakeholder group membership and voting outcomes for each criterion, relevance, comprehensiveness, and ease of understanding, for each PROM per domain. All p-values exceeded 0.05, indicating no statistically significant associations between stakeholder group membership and voting outcomes (see Supplementary file, Appendix 8).

Based on the survey results, consensus on PRO measure selection was reached for both the Symptom Burden and Impacts of Disease and Treatment domains in Round 1, precluding the need for Round 2. The finalised list of measure for the PRO-CAR-T ePRO system were: Symptom Burden Questionnaire™ (Symptom Burden domain), Quality of Life in Cancer Survivors (Impacts of cancer and CAR T-cell treatment), single item FACT-GP5 (Tolerability), and EQ5D-5L (Health-Related Quality of Life).

A description of the selected measures is included in the Supplemental Material, Appendix 6. None of the selected PRO measures had evidence of validation in the target patient population, likely due to the novelty of CAR T-cell therapy. Free text comments related to the relevance of the candidate PRO measures to CAR T-cell therapy and whether there is a requirement to tailor measures to the acute, post-acute and long-term monitoring phases of treatment.

## Discussion

We understand this study to be the first study to use consensus methods to select PRO measures for symptom and quality of life monitoring in the clinical care of patients with haematological malignancies receiving CAR T-cell therapies. The SBQ and QLACS questionnaires were selected by consensus to measure the Symptom Burden and Wider Impacts domains along with the EQ5D-5L, as a generic measure of health-related quality of life, and the single item FACT-GP5 as a measure of treatment tolerability The SBQ and QLACS were selected by Delphi panel members as the PRO measures that were most relevant, comprehensive, and easy to understand whilst the EQ5D-5L and FACT-GP5 were selected by the study team due to their widespread use. Their inclusion in a digital system for remote monitoring of patients following CAR T-cell therapy aims to address the need for acute symptom monitoring and longer-term follow-up arising from the unique and potentially severe toxicity profiles and long-term effects of these therapies.

There is a growing body of literature examining the use of PROs with CAR T-cell therapies and, more generally, advanced therapies.^1^^,^^2^ A core outcome set identified a number of PROs for CAR T-cell clinical trials but stopped short of measure recommendations, instead specifying “red flag” criteria for the included symptoms.^34^ A recent systematic review found few PRO measures developed and validated for adoptive cell therapies generally and for CAR T-cell therapies specifically.^17^ In our study, only one CAR-T-specific PRO measure, the MDASI-CAR, was identified. Whilst the MDASI-CAR was a clear candidate for selection as the only PRO measure with evidence of psychometric evaluation with CAR-T patients, it was excluded in Round 1 as participants’ ratings of relevance and comprehensiveness did not meet the pre-specified thresholds for agreement. Given the lack of PRO measures with evidence of validation in the population of CAR T-cell therapy patients, consensus building methods, such as this modified Delphi study, become critical to establishing the content validity of measures intended for use with CAR T-cell therapy recipients. However, with a paucity of evidence, uncertainty regarding the performance of these measures remains. Psychometric studies to establish the measurement characteristics of the included measures for the CAR-T patient population and qualitative studies to explore the clinical utility and feasibility of the ePRO system are now needed.

A combination of PRO measures ensures relevant concepts are evaluated; however, inclusion of multiple measures must be balanced with the potential burden this places on patients. We note that patients are often willing to complete longer questionnaires if the content is perceived as highly relevant to their experience.^35^ The estimated completion time for the four PRO measures included in the PRO-CAR-T system ranges from 10 to 15 min and will be confirmed in future usability testing.

Numerous toxicity grading systems are available for CAR T-cell-related toxicity, CRS, and ICANS. These systems, such those developed by the American Society for Blood and Bone Marrow Transplantation (ASBMT), the American Society for Transplantation and Cellular Therapist (ASTCT), the National Cancer Institute (NCI) Common Terminology Criteria for Adverse Events (CTCAE), and the CAR-T-Cell Therapy-Associated Toxicity (CARTOX) working group, aim to standardise grading, support toxicity assessment and management, relying on clinician-reported outcomes or performance-based outcomes for toxicity grading.^36^ However, evidence suggests clinicians consistently underreport the incidence and severity of adverse-event related toxicities experienced by patients with patients reporting adverse events earlier and more frequently.^37^^,^^38^ The integration of patient-reported outcomes (PROs) through electronic collection across the care continuum has potential to provide crucial insights into the patient experience of disease and treatment when used alongside clinical assessments. PROs can inform treatment optimization, patient care strategies, and health policy decisions by enabling more accurate assessment of the real-world effectiveness and tolerability of CAR T-cell therapies. Moreover, given the significant symptom burden associated with CAR T-cell therapy and the requirement for clinical teams to monitor patients for 15 years post-treatment, PROs, as real-world evidence and source of registry data, could play a valuable role in understanding the long-term and late effects of CAR T-cell therapies.^39^

As yet, few PROs have been validated in the CAR T-cell therapy patient population. As content validity is considered a PRO measure's most important property, the consensus-based approach used in this study to evaluate the included measures for relevance and comprehensiveness and the use of statistical tests to confirm response stability across groups provides new evidence of the included PRO measures' content validity in this population and is a strength of this study.^21^ However, as the field of CAR T-cell therapies evolves and PRO measures are developed and validated for use with CAR T-cell patients, updates to this review will be necessary to ensure the best available measures are selected.

There are limitations associated with this study. Round 1 survey was complex and required participants to review the instrument cards and questionnaire forms for each of the PRO measures. We were open about the time requirement, stressing that the survey aim was to gather personal/professional opinions and that there were no incorrect responses. Nonetheless, the complexity of the survey and the time required to review the PRO measures may have discouraged some participants, who may have felt they did not have the time to commit to completing the survey or the necessary experience or knowledge.

Consensus was reached after Round 1 with a single measure meeting all three criteria (relevance, comprehensiveness, ease) for each domain. It is plausible that in Round 2 that participants’ ratings could have shifted following discussions, a known phenomenon in Delphi studies.^40^ However, given that consensus was reached in Round 1 according to prespecified stopping criteria, we accepted the panel ratings and concluded that consensus was achieved.

A final limitation relates to the representativeness of the study sample, particularly regarding sample size and participant characteristics. The response rate for the survey was 33% which is consistent with other studies using survey methodology.^41^^,^^42^ There is no consensus on recommended sample sizes for Delphi studies with wide variation reported in the published literature. While it is possible that a larger sample may have yielded different results, a recent narrative review suggested that for highly specialized topics, a Delphi panel of 8–23 participants, with a minimum of 5 participants per subgroup is acceptable.^43^^,^^44^ In terms of response stability in small samples, Akins et al. examined the response characteristics of a small Delphi panel using bootstrapping methods for data expansion. Their findings suggested stable outcomes can be achieved with a relatively small samples, a finding of relevance to highly specialized fields, such as cellular therapies, where the population of available experts is limited.^45^ Sample diversity is also a study limitation. Despite recruiting from a regional CAR T-cell therapy centre situated in an ethnically diverse region of the UK, most participants were of White ethnicity. The lack of diversity amongst patient participants may be a reflection of known health inequalities in accessing CAR T-cell therapies and issues relating to the involvement of ethnic minority communities in health research.^46^, ^47^, ^48^ Confirmation of measure selection in future studies involving representative samples of the CAR-T patient population will provide further evidence of the selected measures’ content validity.

Further research is now needed to validate the selected PRO measures in the CAR-T patient population and explore the generalisability of the study findings beyond the UK's National Health Service. Research is also required to establish the frequency, timing and feasibility for ePRO administration in the context of the care pathway and to ensure barriers to inclusivity and equitable use of these measures within an ePRO system are overcome.^35^^,^^49^ Qualitative research with stakeholders will facilitate co-design of the PRO-CAR-T system, including determining optimal timepoints and frequency of PRO data collection, and clinical alert functionality. Without effective real-time alerts that notify clinical teams when serious side effects occur, ePRO systems for CAR-T cell therapies will not be able to deliver their promise of effective, timely care.^50^

The current study used a modified Delphi method to identify PRO measures to include in an electronic PRO (ePRO) system for monitoring adverse events and quality of life in blood cancer patients receiving CAR T-cell therapies. Use of consensus-building methods for measure selection ensured rigor and transparency, with content validity of the system confirmed by collecting the views of patients, their family members, and health care professionals. To our knowledge, it is one of the first studies to select PRO measures for CAR T-cell therapies based on stakeholder views and represents an important step in the development of co-designed tools to support clinical management of this growing patient population. With successful implementation, ePRO systems for CAR T-cell therapy could improve the patient experience through enhanced monitoring and support the development of more sustainable and affordable pathways, thus enabling more patients to benefit from these novel therapies.

## Contributors

Funding acquisition: MC, AD, CC, FAMK, RC.

Study concept and design: SH, MC, DB, FAMK, CC, CM, AD, EHD, PF, RL, EC, JA.

Data acquisition: SH, FK, RL, PF, KLS.

Data analysis: SH, FK.

Data interpretation: SH, MC, FK, CM, OLA.

Drafting the original manuscript: SH, FK.

Project administration: AW, MC, SH.

Editing and/or critical revision of the manuscript for important intellectual content: All authors.

SH, FK, and RL had access to and verified the underlying data.

All authors read and approved the final version of the manuscript.

## Data sharing statement

The data supporting the findings of this study will be made available upon reasonable written request to the study team. Requests for data access should be directed to: btru@contacts.bham.ac.uk. Data will be provided to researchers whose proposed use of the data has been approved by the study team, with the intention of achieving the goals of the original research. All data shared will be de-identified to ensure participant confidentiality.

## Declaration of generative AI and AI-assisted technologies in the writing process

During the preparation of this work the authors used ChatGPT-4 by OpenAI to check grammar and improve the readability of the text. After using this tool/service, the authors reviewed and edited the content as needed and take full responsibility for the content of the published article.

## Declaration of interests

**SEH** receives funding from the National Institute of Health and Care Research (NIHR), NIHR Blood and Transplant Research Unit (BTRU) in Precision Transplant and Cellular Therapeutics, NIHR Birmingham Biomedical Research Centre (BRC), NIHR Applied Research Collaboration (ARC) West Midlands, UKRI, and UK SPINE. She declares personal fees from Cochlear, Pfizer, Rinri Therapeutics, Astra Zeneca, Aparito (Eli Lilly), and CIS Oncology outside the submitted work.

**MJC** is Director of the Birmingham Health Partners Centre for Regulatory Science and Innovation, Director of the Centre for the Centre for Patient-Reported Outcomes Research and is an NIHR Senior Investigator. MJC receives funding from the NIHR, UKRI, NIHR Birmingham BRC, NIHR ARC West Midlands, European Regional Development Fund, Innovate UK (part of UKRI), LifeArc, Macmillan Cancer Support, UCB Pharma, Janssen, GSK and Gilead. MJC has received personal fees from Astellas, Aparito (Eli Lilly), Boehringer Ingelheim, CIS Oncology, Daiichi Sankyo, Gilead, GSK, Halfloop, ICON, Merck, the Patient-Centred Outcomes Research Institute (PCORI), Pfizer, Takeda, and Vertex outside the submitted work. In addition, a family member owns shares in GSK.

**OLA** receives funding from the NIHR Birmingham BRC, the NIHR Blood and Transplant Research Unit (BTRU) in Precision Transplant and Cellular Therapeutics, NIHR Applied Research Collaboration (ARC) West Midlands, UKRI, Health Foundation, Merck, Gilead, Anthony Nolan, Sarcoma UK, and GSK. He declares personal fees from Gilead Sciences Ltd, Merck, and GSK outside the submitted work.

**CM** receives funding from NIHR Surgical Reconstruction and Microbiology Research Centre (SRMRC), UKRI, National Institute of Health and Care Research (NIHR), NIHR Blood and Transplant Research Unit (BTRU) in Precision Transplant and Cellular Therapeutics, and declares personal fees from Aparito (Eli Lilly) Ltd outside the submitted work.

**FK** receives funding from the National Institute of Health and Care Research (NIHR), NIHR Blood and Transplant Research Unit (BTRU) in Precision Transplant and Cellular Therapeutics and the NIHR Birmingham Biomedical Research Centre (BRC).

**FAMK** receives research funding from Gilead, and personal fees from Therakos, Sanofi, and Vertex outside the submitted work.

**KLS** receives funding from the NIHR Blood BTRU in Precision Cellular Therapeutics.

**EHF** has financial/non-financial interest with Aparito is a wholly owned subsidiary of Eli Lilly and Company.

**DB** received personal payment as consulting fees, payment or honoraria for lectures, presentations, speakers bureaus, manuscript writing or educational events, and support for attending meetings and/or travel from Kite-Gilead.

**CC** is a co-applicant on National Institute of Health and Care Research (NIHR) Blood and Transplant Research Unit in Precision Cellular Therapeutics, received funding from Celgene/Bristol Myers Squibb, NHSBT and Anthony Nolan as Lead applicant, Pfizer Limited as co-applicant, Cancer research UK as co-applicant on 3 projects, Celgene International II as a lead applicant, Acerta Pharma B V as co applicant. CC had received speaker/advisory board fees from Abbvie, Astellas, Daiichi Sankyo, Jazz, Kite, Novartis, and Roche.

**RC** had received consulting fees from Novartis Advisory Board and Accelerating Clinical Trials Ltd.

No other disclosures were reported.

## Appendix ASupplementary data

Appendices 1–8

## References

[1] Efficace F., Cannella L., Sparano F. (2022). Chimeric antigen receptor T-cell therapy in hematologic malignancies and patient-reported outcomes: a scoping review. Hemasphere.

[2] Neelapu S.S., Tummala S., Kebriaei P. (2018). Chimeric antigen receptor T-cell therapy - assessment and management of toxicities. Nat Rev Clin Oncol.

[3] Buitrago J., Adkins S., Hawkins M., Iyamu K., Oort T. (2019). Adult survivorship: considerations following CAR T-cell therapy. Clin J Oncol Nurs.

[4] Puckrin R., Jamani K., Jimenez-Zepeda V.H. (2023). Long-term survivorship care after CAR-T cell therapy. Eur J Haematol.

[5] FDA (2020). https://www.fda.gov/regulatory-information/search-fda-guidance-documents/qualification-process-drug-development-tools-guidance-industry-and-fda-staff.

[6] Commissioner (2021). https://www.fda.gov/regulatory-information/search-fda-guidance-documents/core-patient-reported-outcomes-cancer-clinical-trials.

[7] Basch E., Deal A.M., Kris M.G. (2016). Symptom monitoring with patient-reported outcomes during routine cancer treatment: a randomized controlled trial. J Clin Oncol.

[8] Knight J.M., Szabo A., Arapi I. (2022). Patient-reported outcomes and neurotoxicity markers in patients treated with bispecific LV20.19 CAR T cell therapy. Commun Med.

[9] Gatwood K., Mahmoudjafari Z., Baer B. (2024). Outpatient CAR T-cell therapy as standard of care: current perspectives and considerations. Clin Hematol Int.

[10] Kamal M., Joseph J., Greenbaum U. (2021). Patient-reported outcomes for cancer patients with hematological malignancies undergoing chimeric antigen receptor T cell therapy: a systematic review. Transpl Cell Ther.

[11] Lai-Kwon J., Thorner E., Rutherford C., Crossnohere N., Brundage M. (2024). Integrating patient-reported outcomes into the care of people with advanced cancer—a practical guide. Am Soc Clin Oncol Educ Book.

[12] Hughes S.E., McMullan C., Aiyegbusi O.L. (2024). Protocol for a mixed-methods study to develop and feasibility test a digital system for the capture of patient-reported outcomes (PROs) in patients receiving chimeric antigen receptor T-cell (CAR-T) therapies (the PRO-CAR-T study). BMJ Open.

[13] Khatsuria F., McMullan C., Aiyegbusi O.L. (2024). Development of a conceptual framework for an electronic patient-reported outcome (ePRO) system measuring symptoms and impacts of CAR T-cell therapies in patients with haematological malignancies. Lancet Oncol.

[14] FDA (2022). https://www.fda.gov/regulatory-information/search-fda-guidance-documents/patient-focused-drug-development-selecting-developing-or-modifying-fit-purpose-clinical-outcome.

[15] Peipert J.D., Shaunfield S., Kaiser K. (2022). How do patients interpret and respond to a single-item global indicator of cancer treatment tolerability?. Support Care Cancer.

[16] NICE (2019). https://www.nice.org.uk/about/what-we-do/our-programmes/nice-guidance/technology-appraisal-guidance/eq-5d-5l.

[17] Taylor S., Law K., Coomber-Moore J. (2023). Patient-reported outcome (PRO) instruments used in patients undergoing adoptive cell therapy (ACT) for the treatment of cancer: a systematic review. Syst Rev.

[18] McMullan C., Retzer A., Hughes S.E. (2023). Development and usability testing of an electronic patient-reported outcome (ePRO) solution for patients with inflammatory diseases in an Advanced Therapy Medicinal Product (ATMP) basket trial. J Patient Rep Outcomes.

[19] Gorst S.L., Seylanova N., Dodd S.R. (2023). Core outcome measurement instruments for use in clinical and research settings for adults with post-COVID-19 condition: an international Delphi consensus study. Lancet Respir Med.

[20] Williamson P.R., Altman D.G., Bagley H. (2017). The COMET Handbook: version 1.0. Trials.

[21] Terwee C.B., Prinsen C.A., Chiarotto A. (2017). https://www.cosmin.nl/wp-content/uploads/COSMIN-methodology-for-content-validity-user-manual-v1.pdf.

[22] Barrett D., Heale R. (2020). What are Delphi studies?. Evid Base Nurs.

[23] Taze D., Hartley C., Morgan A.W., Chakrabarty A., Mackie S.L., Griffin K.J. (2022). Developing consensus in Histopathology: the role of the Delphi method. Histopathology.

[24] Vogel C., Zwolinsky S., Griffiths C., Hobbs M., Henderson E., Wilkins E. (2019). A Delphi study to build consensus on the definition and use of big data in obesity research. Int J Obes.

[25] Murphy M., Hollinghurst S., Salisbury C. (2016). Agreeing the content of a patient-reported outcome measure for primary care: a Delphi consensus study. Health Expect.

[26] Hughes S.E., Haroon S., Subramanian A. (2022). Development and validation of the symptom burden questionnaire for long covid (SBQ-LC): rasch analysis. BMJ.

[27] McQuellon R.P., Russell G.B., Cella D.F. (1997). Quality of life measurement in bone marrow transplantation: development of the Functional Assessment of Cancer Therapy-Bone Marrow Transplant (FACT-BMT) scale. Bone Marrow Transplant.

[28] Avis N.E., Smith K.W., McGraw S., Smith R.G., Petronis V.M., Carver C.S. (2005). Assessing quality of life in adult cancer survivors (QLACS). Qual Life Res.

[29] Boyes A., Girgis A., Lecathelinais C. (2009). Brief assessment of adult cancer patients' perceived needs: development and validation of the 34-item Supportive Care Needs Survey (SCNS-SF34). J Eval Clin Pract.

[30] Grant M., Ferrell B., Schmidt G.M., Fonbuena P., Niland J.C., Forman S.J. (1992). Measurement of quality of life in bone marrow transplantation survivors. Qual Life Res.

[31] Osborne T.R., Ramsenthaler C., Schey S.A., Siegert R.J., Edmonds P.M., Higginson I.J. (2015). Improving the assessment of quality of life in the clinical care of myeloma patients: the development and validation of the Myeloma Patient Outcome Scale (MyPOS). BMC Cancer.

[32] Wang X.S., Srour S.A., Mendoza T. (2023). Development and validation of a patient-reported outcome measure to assess symptom burden after chimeric antigen receptor T-cell therapy. Br J Haematol.

[33] Kluijtmans M., de Haan E., Akkerman S., van Tartwijk J. (2017). Professional identity in clinician-scientists: brokers between care and science. Med Educ.

[34] Spanjaart A.M., Pennings E.R.A., Kos M. (2023). Development of a core set of patient- and caregiver-reported signs and symptoms to facilitate early recognition of acute chimeric antigen receptor T-cell therapy toxicities. JCO Oncology Practice.

[35] Aiyegbusi O.L., Cruz Rivera S., Roydhouse J. (2024). Recommendations to address respondent burden associated with patient-reported outcome assessment. Nat Med.

[36] Pennisi M., Jain T., Santomasso B.D. (2020). Comparing CAR T-cell toxicity grading systems: application of the ASTCT grading system and implications for management. Blood Adv.

[37] Atkinson T.M., Ryan S.J., Bennett A.V. (2016). The association between clinician-based common terminology criteria for adverse events (CTCAE) and patient-reported outcomes (PRO): a systematic review. Support Care Cancer.

[38] Basch E., Jia X., Heller G. (2009). Adverse symptom event reporting by patients vs clinicians: relationships with clinical outcomes. J Natl Cancer Inst.

[39] UK Stem Cell Strategic Forum. A ten year vision for stem cell transplantation and cellular therapies. NHS Blood and Transplant. https://www.nhsbt.nhs.uk/who-we-are/performance-and-strategy/stem-cell-and-advanced-cellular-therapy-strategy/.

[40] Dajani J.S., Sincoff M.Z., Talley W.K. (1979). Stability and agreement criteria for the termination of Delphi studies. Technol Forecast Soc Change.

[41] Lindemann N. Average survey response rate 2021. https://pointerpro.com/blog/average-survey-response-rate/.

[42] Morton S.M.B., Bandara D.K., Robinson E.M., Carr P.E.A. (2012). In the 21st Century, what is an acceptable response rate?. Aust N Z J Publ Health.

[43] Belton I., MacDonald A., Wright G., Hamlin I. (2019). Improving the practical application of the Delphi method in group-based judgment: a six-step prescription for a well-founded and defensible process. Technol Forecast Soc Change.

[44] Shang Z. (2023). Use of Delphi in health sciences research: a narrative review. Medicine.

[45] Akins R.B., Tolson H., Cole B.R. (2005). Stability of response characteristics of a Delphi panel: application of bootstrap data expansion. BMC Med Res Methodol.

[46] Smart A., Harrison E. (2016). The under-representation of minority ethnic groups in UK medical research. Ethn Health.

[47] Ahmed N., Shahzad M., Shippey E. (2022). Socioeconomic and racial disparity in chimeric antigen receptor T cell therapy access. Transpl Cell Ther.

[48] Ahmed N., Sun F., Teigland C. (2024). Chimeric antigen receptor T-cell access in patients with relapsed/refractory large B-cell lymphoma: association of access with social determinants of health and travel time to treatment centers. Transpl Cell Ther.

[49] Calvert M.J., Cruz Rivera S., Retzer A. (2022). Patient reported outcome assessment must be inclusive and equitable. Nat Med.

[50] Kyte D., Draper H., Calvert M. (2013). Patient-reported outcome alerts. JAMA.

