# Supplementary material for: Measure selection for an electronic patient-reported outcome (ePRO) system for CAR T-cell therapy patients: a modified Delphi consensus study
Source: eClinicalMedicine. 2025 May 28;84:103256. doi: 10.1016/j.eclinm.2025.103256 (PMC12159924; doi:10.1016/j.eclinm.2025.103256)
Supplement: Appendices 1–8 [file mmc1.pdf]

Supplementary Appendices: Hughes, SE et al. Measure selection for an electronic patient-reported outcome (ePRO) system for CAR-T cell therapy patients: a modified Delphi consensus study.

Table of Contents

Appendix 1: Schematic domain framework* and measurement model for the PRO-CAR-T ePRO System)[1] 2
Appendix 2: Search strategies...3
Appendix 3: Results of concept mapping 11
Appendix 4: Eligibility criteria applied to shortlisting of PRO measures for inclusion in the Delphi survey.......17
Appendix 5: Evidence of psychometric evaluation of the candidate PRO measures' measurement properties** .18
Appendix 6: Instrument cards for the shortlisted PRO measures 19
Appendix 7: Example screenshots from Delphi Round 1 Online Survey.......................................26
Appendix 8: Results of Fisher's exact test showing exact probability values for each of the candidate PROMs for the indicators of relevance, comprehensiveness and ease of understanding per domain.................................29
References 30

Appendix 1: Schematic domain framework* and measurement model for the PRO-CAR-T ePRO System)[1]

PRO-CAR-T™ Study

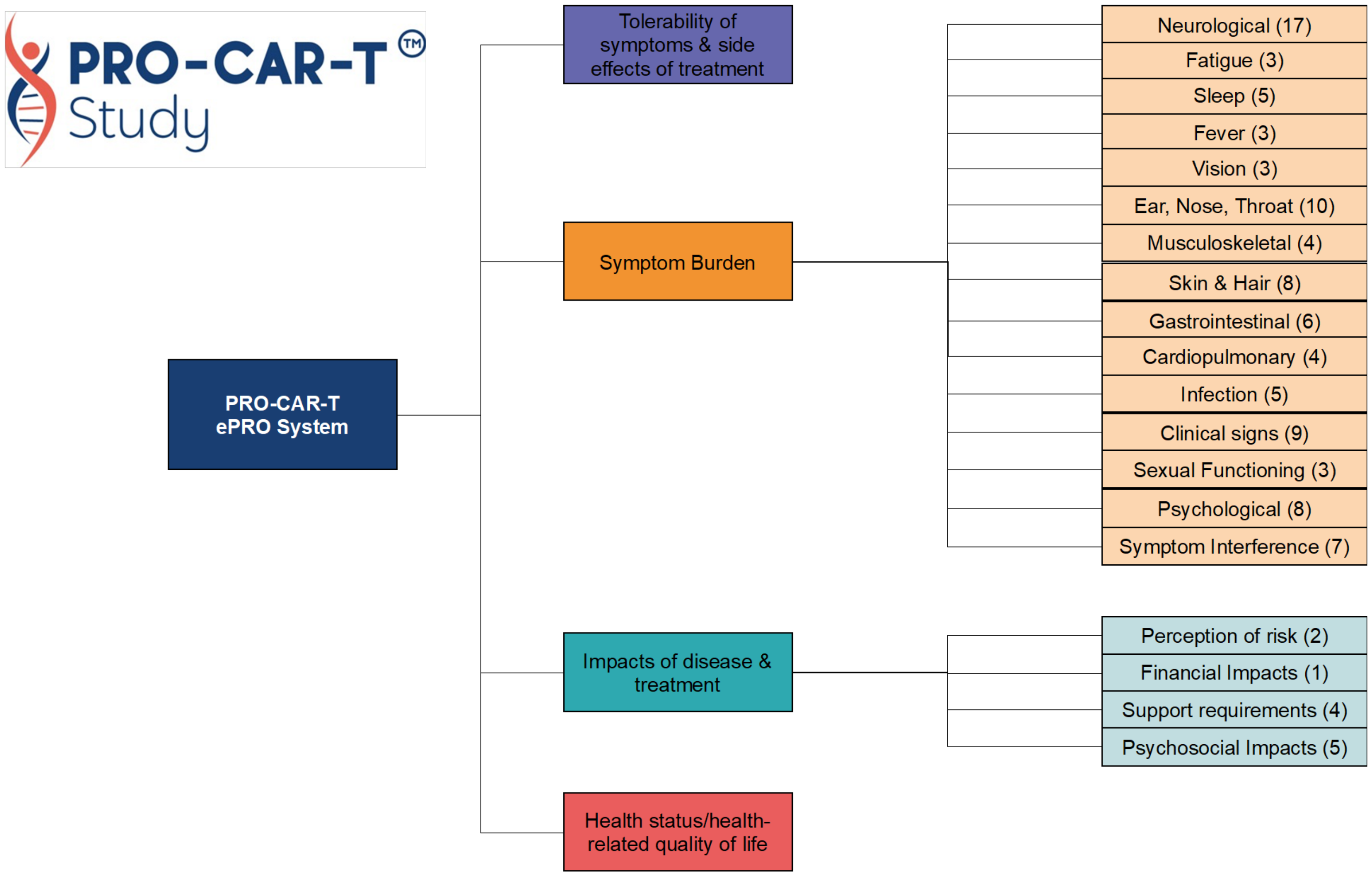

* Numbers in parentheses denote the number of concepts included in each subdomain.

Hughes et al. Measure selection for an electronic patient-reported outcome (ePRO) system for CAR T-cell therapy patients: a modified Delphi consensus study

Appendix 2: Search strategies

Table 2.1: Databases and information sources searched to locate candidate PRO measures.

Source	Methods	Searches and terminology
Rapid review[1]	PRO measures of symptom burden and quality of life were identified from studies included in the rapid review[1]	See Supplementary file for search strategy and results[1]
MEDLINE<br>Google	Targeted searches were conducted on MEDLINE and Google to identify PRO measures covering constructs included in the Impacts and Diseases Treatment domain,<br><br>MEDLINE search strategy was adapted from search strategies described in Aiyegbusi et al. including the Oxford PROM filter[2] (Note 1.1). we used the search terms mentioned in table 1.1 for all the sub domains in combination with the Oxford PROM filter to search for domain specific PROMs available on MEDLINE.<br><br>We conducted a Google search using the "concept" terms described in table 1.1 in combination with "patient-reported outcome". The first 25 results were screened for PRO measures.	Note 2.1, Table 2.2
COSMIN database of systematic reviews[3]	The COSMIN database of systematic reviews was searched for all cancer-related or generic systematic reviews describing development, evaluation or validation of the PRO measures for constructs included in the Impacts of Disease and Treatment domain.	Table 2.3, 2.4

Table 2.2: Search terms per sub-domain of the Impacts of Disease and Treatment domain included in the MEDLINE searches

Sub-domain	Concept	Synonyms (MESH headings etc) with BOOLEAN "or"
Psychosocial impacts	Access to resources (social determinants of health)<br>Social withdrawal/social functioning/ social wellbeing<br>fear and isolated (confined and being away from family)<br>feeling burden to my family<br>feeling of confinement<br>Access to resources (social determinants of health)	• Psychosocial<br>• psychosocial factor*<br>• Psychosocial aspect*<br>• Psycho* Onco*<br>• Health adj3 social determinant*<br>• Social isolation<br>• Lonel*<br>• Social Well*<br>• Family adj4 burden<br>• (feel* adj3 confine*) OR (feel* adj3 isolate) OR (Feel* adj3 burden)<br>• (stress adj3 confine*) OR (stress adj3 isolate) OR (stress adj3 burden)<br>• (pressure adj3 confine*) OR (pressure adj3 isolate) OR (pressure adj3 burden)<br>• Health adj3 structural determinant*<br>• Social* detach*<br>• Social* withdraw*<br>• (fear* adj3 confine*) OR (fear* adj3 isolate) OR (Fear* adj3 burden)<br>• (trepidat* adj3 confine*) OR (trepidat* adj3 isolate) OR (trepidat* adj3 burden)<br>• (apprehens* adj3 confine*) OR (apprehens* adj3 isolate)
Tolerability	Nature/amount/intensity of side effects	• Tolerab*<br>• (Tolerab* adj4 side effect*) OR (tolerab* adj4 adverse event*) OR (tolerab* adj4 aftereffect*) OR (tolerab* adj4 repercussion*)<br>• (Intensity adj3 side effect*) OR (intensity adj3 adverse event*) OR (intensity adj4 aftereffect*) OR (intensity adj4 repercussion*)<br>• (bear* adj3 side effect*) OR (bear* adj3 adverse event*) OR (bear* adj4 aftereffect*) OR (bear* adj4 repercussion*)<br>• (liveab* adj3 side effect*) OR (liveab* adj3 adverse event*) OR (liveab* adj4 aftereffect*) OR (liveab* adj4 repercussion*)

Sub-domain	Concept	Synonyms (MESH headings etc) with BOOLEAN “or”
Perception of risk	Fear /worry of COVID-19	• Perception of risk<br>• (Process* risk adj3 disease*) OR (Process* risk adj3 infection) OR (process* risk adj3 infestation*)<br>• Risk adj2 aversion<br>• Treatment adj2 aversion<br>• Risk adj2 apperihens*<br>• Risk adj2 trepidat*<br>• Treatment adj2 trepidat*<br>• Treatment adj2 apprehens*<br>• Risk attitude*<br>• (Fear* adj3 infection*) OR (Fear* ad3 infestation*) OR (Fear* adj3 disease*)<br>• (Fear* adj3 progression) OR (Fear* adj3 relapse)<br>• (worr* adj3 infection*) OR (worr* ad3 infestation*) OR (worr* adj3 disease*)<br>• (worr* adj3 progression) OR (worr* adj3 relapse)<br>• (wary adj3 infection*) OR (wary ad3 infestation*) OR (wary adj3 disease*)<br>• (wary adj3 progression) OR (wary adj3 relapse)<br>• (trepidat* adj3 infection*) OR (trepidat* ad3 infestation*) OR (trepidat* adj3 disease*)<br>• (trepidat* adj3 progression) OR (trepidat* adj3 relapse)<br>• (apprehens* adj3 infection*) OR (apprehens* ad3 infestation*) OR (apprehens* adj3 disease*)<br>• (apprehens* adj3 progression) OR (apprehens* adj3 relapse)<br>• (fear* adj4 COVID) OR (worr* adj4 COVID) OR (wary adj4 COVID) OR (trepidat* adj4 COVID) OR (apprehens* adj4 COVID)
Financial Impacts	Financial burden of treatment	• Financial* Stress*<br>• Stress*, Financial<br>• Financial Pressure*<br>• Pressure*, Financial

Sub-domain	Concept	Synonyms (MESH headings etc) with BOOLEAN "or"
		• Financial Toxicity<br>• Financial Toxicities<br>• Toxicities, Financial<br>• Toxicity, Financial<br>• Challenge*, Financial<br>• Financial Challenge*<br>• Economic Burden<br>• Burden*, Economic<br>• Financial Burden*<br>• Burden*, Financial<br>• Financial Burdens<br>• Financial Hardship*<br>• Hardship*, Financial<br>• Economic Hardship*<br>• Hardship*, Economic
Support requirements	Reliance on authoritative medical personnel	• Support requir*<br>• (Rel* adj4 medical person*) OR (Depend* adj4 medical person*) OR (support adj4 medical person*)
	Spiritual need	• (requir* adj4 spiritual need*) OR (rel* adj4 spiritual need*) OR (support adj4 spiritual need*)<br>• Religious support<br>• Religious need<br>• (requir* adj4 religious support) OR (rel* adj4 religious support) OR (support adj4 religious support)<br>• Spiritual strength<br>• Spiritual support
	Information need	• Support* information*<br>• support adj3 information source*<br>• Support adj3 data source*<br>• Data source*<br>• Information source*

**Sub-domain**	**Concept**	**Synonyms (MESH headings etc) with BOOLEAN "or"**
	Social support	• Social support*<br>• (Social support* adj3 perceived)<br>• Perceived social support*<br>• Online adj2 social support*
Quality of life	Quality of life	• (Health adj (relate* adj (quality adj (of adj (life)))))<br>• Hrqol<br>• Life adj quality<br>• Quality of life<br>• Adjust* adj3 Qol<br>• Qol<br>• Adjust* adj3 life quality<br>• Adjust* adj3 hrqol<br>• Adjust* adj3 (Health adj (relate* adj (quality adj (of adj (life)))))<br>• Adjust* adj3 (quality adj (of adj (life)))

Hughes et al. Measure selection for an electronic patient-reported outcome (ePRO) system for CAR T-cell therapy patients: a modified Delphi consensus study

Note 2.1: MEDLINE search strategy (based on the search strategy presented in Aiyegbusi et al. which includes the Oxford PROM filter)[2]

(HR-PRO or HRPRO or HRQL or HRQoL or QL or QoL).ti,ab.

quality of life.mp.

(health index* or health indices or health profile*).ti,ab.

health status.mp.

((patient or self or child or parent or carer or proxy) adj (appraisal* or appraised or report or reported or reporting or rated or rating or based or assessed or assessment*)).ti,ab.

((disability or function or functional or functions or subjective or utility or utilities or wellbeing or well being) adj2 (index or indices or instrument or instruments or measure or measures or questionnaire* or profile or profiles or scale or scales or score or scores or status or survey or surveys)).ti,ab.

((((patient adj reported adj outcome adj measure*) or patient) adj reported adj outcome*) or capability or capabilities).mp.

1 or 2 or 3 or 4 or 5 or 6 or 7

Psychosocial Functioning/ or Psychosocial Support Systems/

psychosocial factor*.ti,ab.

psychosocial aspect*.ti,ab.

psychosocial impact*.ti,ab.

psycho* onco*.ti,ab.

(Health adj3 social determinant*).ti,ab.

social isolation.ti,ab.

lonel*.ti,ab.

social well*.ti,ab.

(Family adj4 burden).ti,ab.

((feel* adj3 confine*) or (feel* adj3 isolate) or (Feel* adj3 burden)).ti,ab.

((stress adj3 confine*) or (stress adj3 isolate) or (stress adj3 burden)).ti,ab.

((pressure adj3 confine*) or (pressure adj3 isolate) or (pressure adj3 burden)).ti,ab.

(Health adj3 structural determinant*).ti,ab.

Social* detach*.ti,ab.

Social* withdraw*.ti,ab.

((fear* adj3 confine*) or (fear* adj3 isolate) or (Fear* adj3 burden)).ti,ab.

((trepidat* adj3 confine*) or (trepidat* adj3 isolate) or (trepidat* adj3 burden)).ti,ab.

((apprehens* adj3 confine*) or (apprehens* adj3 isolate)).ti,ab.

9 or 10 or 11 or 12 or 13 or 14 or 15 or 16 or 17 or 18 or 19 or 20 or 21 or 22 or 23 or 24 or 25 or 26 or 27

(((((((((((((((((((((((((((((Instrumentation or method* or Validation Studies or Comparative Study).mp. or psychometrics/ or psychometr*.mp. or clinimetr*.mp. or clinometr*.mp. or outcome assessment health care/ or outcome assessment*.ti,ab. or outcome measure*.mp. or observer variation/ or observer variation*.ti,ab. or Health Status Indicators/ or reproducibility of results/ or reproducib*.ti,ab. or discriminant analysis/ or reliab*.ti,ab. or unreliab*.ti,ab. or valid*.ti,ab. or coefficient of variation.ti,ab. or coefficient*.ti,ab. or homogeneity.ti,ab. or homogeneous.ti,ab. or internal consistency.ti,ab. or cronbach*.ti,ab.) and alpha*.ti,ab.) or item*.ti,ab.) and correlation*.ti,ab.) or selection*.ti,ab. or reduction*.ti,ab. or agreement.mp. or precision.mp. or imprecision.mp. or precise value*.mp. or test-retest.ti,ab. or test.ti,ab.) and retest.ti,ab.) or reliab*.ti,ab.) and test.ti,ab.) or retest.ti,ab. or stability.ti,ab. or interrater.ti,ab. or inter-rater.ti,ab. or intrarater.ti,ab. or intra-rater.ti,ab. or intertester.ti,ab. or inter-tester.ti,ab. or intratester.ti,ab. or intra- tester.ti,ab. or interobserver.ti,ab. or inter-observer.ti,ab. or intraobserver.ti,ab. or intra-observer.ti,ab. or intertechnician.ti,ab. or inter-technician.ti,ab. or intratechnician.ti,ab. or intra-technician.ti,ab. or interexaminer.ti,ab. or inter- examiner.ti,ab. or intraexaminer.ti,ab. or intra-examiner.ti,ab. or interassay.ti,ab. or inter-assay.ti,ab. or intraassay.ti,ab. or intra-assay.ti,ab. or interindividual.ti,ab. or inter-individual.ti,ab. or intraindividual.ti,ab. or intra-individual.ti,ab. or interparticipant.ti,ab. or inter-participant.ti,ab. or intraparticipant.ti,ab. or intra- participant.ti,ab. or kappa*.ti,ab. or kappa's.ti,ab. or repeatab*.mp. or replicab*.mp. or repeated.mp.) and measure*.mp.) or finding*.mp. or result*.mp. or test*.mp. or generaliza*.ti,ab. or generalisa*.ti,ab. or concordance.ti,ab. or intraclass.ti,ab.) and correlation*.ti,ab.) or discriminative.ti,ab. or known group.ti,ab. or factor analysis.ti,ab. or factor analyses.ti,ab. or factor structure.ti,ab. or factor structure.ti,ab. or dimension*.ti,ab. or subscale*.ti,ab. or multitrait.ti,ab.) and scaling.ti,ab. and analysis.ti,ab.) or analyses.ti,ab. or item discriminant.ti,ab. or interscale correlation*.ti,ab. or error.ti,ab. or errors.ti,ab. or individual variability.ti,ab. or interval variability.ti,ab. or rate variability.ti,ab. or variability.ti,ab.) and analysis.ti,ab.) or value*.ti,ab. or uncertainty.ti,ab.) and measurement.ti,ab.) or measuring.ti,ab. or standard error of measurement.ti,ab. or sensitiv*.ti,ab. or responsive*.ti,ab. or limit*.ti,ab.) and detection.ti,ab.) or minimal detectable concentration.ti,ab. or interpretab*.ti,ab. or minimal.ti,ab. or minimally.ti,ab. or clinical.ti,ab. or clinically.ti,ab.) and important.ti,ab.) or significant.ti,ab. or detectable.ti,ab.) and change.ti,ab.) or difference.ti,ab. or small*.ti,ab.) and real.ti,ab.) or detectable.ti,ab.) and change.ti,ab.) or difference.ti,ab. or meaningful change.ti,ab. or ceiling effect.ti,ab. or floor effect.ti,ab. or Item response model.ti,ab. or IRT.ti,ab. or Rasch.ti,ab. or Differential item functioning.ti,ab. or DIF.ti,ab. or computer adaptive testing.ti,ab. or item bank.ti,ab. or cross-cultural equivalence.ti,ab.

(PRO integration or Clinical PRO application* or telePRO or automated PRO algorithm* or screening purpose* or PRO questionnaire* or Patient-reported outcome questionnaire* or Patient-reported symptom* or Patient-centred care or Patient self- report* or Self-report health or Self-rated health or Self-reported measure* of health or Health outcome* or Health communication* or Hospital performance evaluation* or Automated telephone survey system* or paper-based survey* or web-based survey* or web-based PRO platform* or web-based system* or PRO collection* or PRO measure* or PRO intervention* or PRO assessment intervention* or PRO data or PRO assessment* or Routine PRO assessment* or Routine PRO collection or Symptom assessment* or Symptom monitoring or Symptom data or Functional status or Electronic PRO assessment* or Electronic PRO system* or ePRO or ePRO* or ePRO system* or PRO system* or Generic PRO system* or PRO-based clinical alert system*).mp.

29 or 30

8 and 28 and 31

Table 2.3: Search results for the COSMIN database of systematic reviews of PRO measures.

Search term	Filters	Search results
Social isolation	Without any restrictions	436
Tolerability	Without any restrictions	9
Fear	Without any restrictions	0
Fear and isolation	Without any restrictions	0
Psychosocial impact	Without any restrictions	36
Isolation	Without any restrictions	3
Social isolation measures	With neoplasm and age restrictions	47
Social withdrawal	With similar restrictions	46
Finance	Without restrictions	2
Burden on family	Without restrictions	49
Quality of life	With restrictions (age, neoplasm and PROs)	140
Support	With similar restriction above	32
Total reviews identified		800
Total reviews selected after screening		55

Table 2.4: Eligibility criteria for the selection of systematic reviews of outcome measurement instruments registered with the COSMIN database (see Appendix 4 for eligibility criteria for PRO measures)[3]

**Systematic review screening**	The abstract, results, and discussion section of the identified systematic reviews were screened and included if the following criteria were met:<br>• if the systematic review identified PRO instruments measuring constructs included in the Impact of Disease and Treatment domain of the conceptual framework (Table 1.2)<br>• review was published in English

Appendix 3: Results of concept mapping

Table 3.1: Results of item-level concept mapping of the identified PRO measures to the Impacts of Disease and Treatment, Tolerability, and Health-Related Quality of Life domains of the ePRO framework.

PROM	Overall Conceptual Coverage (21/21) (% coverage)	Interference* (7/7) (% coverage)	Psychosocial impacts (5/5) (% coverage)	Perception of risk (2/2) (% coverage)	Financial Impacts (1/1) (% coverage)	Support requirements (4/4) (% coverage)	Tolerability (1/1)	Quality of Life (1/1)
Needs Assessment for Advance Cancer Patients (NA-ACP)	94·7	100	100	50	100	100	1	0
CAncer Rehabilitation Evaluation System-Short Form (CARES)	89·5	100	100	50	100	75	1	0
Survivor Unmet need survey (SUNS)	84·2	85·7	80	50	100	100	0	1
Functional Assessment of Cancer Therapy – Bone Marrow Transplant general+ Bone marrow Transplant (FACT (G+BMT)	73·7	71·4	80	100	100	50	1	1
Quality Of Life Bone Marrow Transplant Survivors (Qol-BMT)	73·7	85·7	80	50	100	50	0	1
Supportive Cancer Needs Survey – Long Form (SCNS-LF)	68·4	85·7	40	50	100	75	1	0
Myeloma Patient outcome scale (MY-POS)	63·2	71·4	20	100	100	75	1	1
Cancer Problem In Living Scale (CPILS)	63·2	85·7	60	50	100	25	0	0
Supportive Cancer Needs Assessment Tool for Indigenous People (SCNAT-IP)	57·9	42·9	40	50	100	100	1	0
Cancer Needs Questionnaire Short form (CNQ-SF)	57·9	57·1	60	50	0	75	0	0

PROM	Overall Conceptual Coverage (21/21) (% coverage)	Interference* (7/7) (% coverage)	Psychosocial impacts (5/5) (% coverage)	Perception of risk (2/2) (% coverage)	Financial Impacts (1/1) (% coverage)	Support requirements (4/4) (% coverage)	Tolerability (1/1)	Quality of Life (1/1)
European Organisation for Research And Treatment Of Cancer – Quality of Life Core (EORTC QLQ-C30 version 3.0)	52·6	85·7	60		100	0	0	1
Cancer Survivor Unmet Need Measure (CaSUN)	47·4	28·6	20	50	100	100	0	1
Functional Assessment of Cancer Therapy – Bone Marrow Transplant Multiple Myeloma (FACT-MM)	47·4	100	0	100	0	0	1	1
Quality of life Index (QLI)	42·1	42·9	40	0	100	50	0	0
Functional Assessment of Cancer Therapy – Bone Marrow Transplant Lymphoma (FACT-LYM)	36·8	42·9	80	0	0	0	1	1
Optimal Living Profile (OLP)	36·8	42·9	40	0	0	50	0	0
Distress-Recurrence Subscale of the Quality of Life in Adult Cancer Survivors Scale (DRS-QLACS)	36·8	0	80	50	100	25	0	0
Symptom burden questionnaire – Long Covid (SBQ™-LC)	36·8	85·7	20	0	0	0	0	0
European Organisation For Research And Treatment Of Cancer – Core+ Chronic Myeloid Leukaemia (EORTC-QLQ30-CML24)	31·6	28·6	20	50	0	50	0	1
Functional Assessment of Chronic Illness Therapy – Fatigue Scale (FACIT-Fatigue)	31·6	57·1	20	0	0	25	0	1
Functional Assessment of Cancer Therapy – Bone Marrow Transplant general (FACT-G)	31·6	42·9	60	0	0	0	1	1
MD Anderson Symptom Inventory (MDASI)	31·6	85·7	0	0	0	0	0	0
MD Anderson Symptom Inventory – CAR (MDASI-CAR)	31·6	85·7	0	0	0	0	0	0

Hughes et al. Measure selection for an electronic patient-reported outcome (ePRO) system for CAR T-cell therapy patients: a modified Delphi consensus study

PROM	Overall Conceptual Coverage (21/21) (% coverage)	Interference* (7/7) (% coverage)	Psychosocial impacts (5/5) (% coverage)	Perception of risk (2/2) (% coverage)	Financial Impacts (1/1) (% coverage)	Support requirements (4/4) (% coverage)	Tolerability (1/1)	Quality of Life (1/1)
Short Form-36 Health Survey (SF-36)	31·6	71·4	20	0	0	0	0	0
European Organisation For Research And Treatment Of Cancer – Bone Marrow Transplant (EORTC-BMT)	26·3	42·9	20	0	100	0	0	0
Multicultural Quality of Life Index (MQLI)	21·1	28·6	0	0	0	50	0	1
Patient-Reported Outcomes Measurement Information System – Global Health (PROMIS Scale v1.2 Global Health)	21·1	42·9	20	0	0	0	0	1
Perceived Wellness Survey (PWS)	21·1	28·6	20	0	0	25	0	0
Patient-Reported Outcomes Measurement Information System (PROMIS-29)	21·1	57·1	0	0	0	0	0	0
Quality Of Health, Quality Of Life (QAHL)	15·8	28·6	0	1	0	0	0	1

Table 3.2: Results of item-level concept mapping of the identified PRO measures to Symptom Burden domain of the ePRO framework.

PROM	Overall conceptual coverage (88/88)	Overall conceptual coverage (% coverage)	Neurological (% coverage)	Skin and Hair (% coverage)	Ear, Nose & Throat (%coverage)	Signs (% coverage)	Cardiopulmonary (% coverage)	Psychological (% coverage)	Gastrointestinal (% coverage)	Fever-related (% coverage)	Infections (% coverage)	Sleep (% coverage)	Musculoskeletal (% coverage)	Sexual functioning (% coverage)	Fatigue (% coverage)	Vision (%coverage)
**Domain: Symptom Burden**																
Symptom Burden Questionnaire – Long Covid (SBQ™-LC)	48	54·5	47·1	50·0	50·0	66·7	25·0	37·5	100·0	100·0	20·0	60·0	75·0	66·7	33·3	66·7
Patient reported outcomes Common Terminology Criteria for Adverse Events (PRO-CTCAE)	32	36·4	17·6	25·0	30·0	66·7	25·0	37·5	83·3	66·7	20·0	20·0	50·0	0·0	33·3	66·7
Needs Assessment For Advance Cancer Patients (NA-ACP)	29	33·0	17·6	0·0	30·0	55·6	25·0	62·5	66·7	33·3	40·0	40·0	0·0	0·0	33·3	0·0
MD Anderson Inventory CAR (MDASI-CAR)	22	25·0	35·3	0·0	10·0	44·4	25·0	25·0	50·0	33·3	0.0	40.0	0.0	33·3	33·3	0·0
Quality Of Life Bone Marrow Transplant Survivors (QoL-BMT)	18	20·5	11·8	0·0	30·0	11·1	25·0	50·0	33·3	0·0	0·0	20·0	0·0	66·7	33·3	33·3
CAncer Rehabilitation Evaluation System-Short Form (CARES)	17	19·3	11·8	0·0	20·0	33·3	0·0	37·5	50·0	0·0	0·0	20·0	0·0	66·7	0·0	0·0
Gothenburg Quality Of Life Measurement Scale (GQL)	16	18·2	5·9	0·0	0·0	55·6	0·0	12·5	66·7	33·3	0·0	20·0	25·0	0·0	33·3	33·3

PROM	Overall conceptual coverage (88/88)	Overall conceptual coverage (% coverage)	Neurological (% coverage)	Skin and Hair (% coverage)	Ear, Nose & Throat (%coverage)	Signs (% coverage)	Cardiopulmonary (% coverage)	Psychological (% coverage)	Gastrointestinal (% coverage)	Fever-related (% coverage)	Infections (% coverage)	Sleep (% coverage)	Musculoskeletal (% coverage)	Sexual functioning (% coverage)	Fatigue (% coverage)	Vision (%coverage)
EORTC QLQ-C30 version 3·0	14	15·9	11·8	0·0	0·0	11·1	25·0	37·5	66·7	0·0	0·0	20·0	0·0	0·0	66·7	0·0
Myeloma Patient Outcome Scale (MY-POS)	14	15·9	11·8	0·0	10·0	0·0	0·0	37·5	66·7	0·0	20.0	20·0	0·0	33·3	33·3	0·0
Functional Assessment of Cancer Therapy – Lymphoma (FACT-LYM)	13	14·8	5·9	0·0	0·0	33·3	0·0	37·5	16·7	33·3	20·0	40·0	0·0	0·00	33·3	0·0
Functional Assessment of Cancer Therapy General +Bone marrow transplant (FACT(G+BMT))	13	14·8	11·8	0·0	10·0	11·1	25·0	12·5	16·7	0·0	20·0	40·0	0·0	33·3	33·3	33·3
European Organisation For Research And Treatment Of Cancer – Chronic Myeloid Leukaemia (EORTC-QLQ30+CML24)	12	13·6	0·0	0·0	10·0	22·2	0·0	0·0	16·7	33·3	0·0	0·0	50·0	0·0	0·0	0·0
MD Anderson Inventory (MDASI)	12	13·6	11·8	0·0	10·0	11·1	25·0	25·0	33·3	0·0	0·0	40·0	0·0	0·00	33·3	0·0
The Self-Rating Anxiety Scale (SAS)	12	13·6	17·6	0·0	0·0	22·2	25·0	37·5	16·7	0·0	0·0	40·0	0·0	0·00	0·0	0·0
Distress-Recurrence Subscale of the Quality of Life in Adult Cancer	11	12·5	17·6	0·0	0·0	11·1	0·0	37·5	0·0	0·0	0·0	0·0	0·0	66·7	33·3	0·0

PROM	Overall conceptual coverage (88/88)	Overall conceptual coverage (% coverage)	Neurological (% coverage)	Skin and Hair (% coverage)	Ear, Nose & Throat (%coverage)	Signs (% coverage)	Cardiopulmonary (% coverage)	Psychological (% coverage)	Gastrointestinal (% coverage)	Fever-related (% coverage)	Infections (% coverage)	Sleep (% coverage)	Musculoskeletal (% coverage)	Sexual functioning (% coverage)	Fatigue (% coverage)	Vision (%coverage)
Survivors Scale (DRS-QLACS)																
Supportive Cancer Needs Survey – Long Form (SCNS-LF)	10	11·4	0·0	0·0	0·0	11·1	0·0	50·0	33·3	0·0	0·0	20·0	0·0	33·3	33·3	0·0
NeuroQoLv2 questionnaire	9	10·2	11·8	0·0	10·0	0·0	0·0	37·5	0·0	0·0	0·0	20·0	0·0	0·33	33·3	0·0
Functional Assessment of Cancer Therapy – Bone Marrow Transplant Multiple Myeloma (FACT-MM)	9	10·2	5·9	0·0	0·0	22·2	0·0	12·5	16·7	0·0	20·0	20·0	25·0	0·0	33·3	0·0
Patient-Reported Outcomes Measurement Information System (PROMIS-29)	9	10·2	0·0	25·0	0·0	11·1	0·0	37·5	0·0	0·0	0·0	40·0	0·0	0·00	33·3	0·0

Hughes et al. Measure selection for an electronic patient-reported outcome (ePRO) system for CAR T-cell therapy patients: a modified Delphi consensus study

Appendix 4: Eligibility criteria applied to shortlisting of PRO measures for inclusion in the Delphi survey

Inclusion criteria	Exclusion criteria
• A) PRO measure provides coverage of ≥70% subdomains within the Symptom Burden domain.<br><br>OR<br><br>B) PRO measure provides full coverage (100%) of the subdomains represented in the Impacts of Disease and Treatment domain.*<br><br>• Suitable for delivery via an ePRO system.<br><br>• Recall period of 7 days or less.<br><br>• Suitable for clinical use.	• A) PRO measure provides <70% subdomain coverage within Symptom Burden domain<br>OR<br>B) <100% coverage of the Impacts of Disease and Treatment domain.<br><br>• PRO measure is not suitable for delivery via an ePRO system<br><br>• PRO measure uses a recall period greater than 7 days.<br><br>• PRO measure not suitable for use in routine clinical practice.<br><br>• Review copy not available.<br><br>• Information on licensing or permission for use not attainable.<br><br>• PRO measure not available in English.

*Coverage defined as having at least one item mapped to a given subdomain.

Hughes et al. Measure selection for an electronic patient-reported outcome (ePRO) system for CAR T-cell therapy patients: a modified Delphi consensus study

Appendix 5: Evidence of psychometric evaluation of the candidate PRO measures' measurement properties**

Measure	Source	Internal consistency reliability	Structural validity	Reliability	Measurement error	Construct validity	Criterion Validity	Responsiveness	Interpretability
QLACS	Avis et al. (2005)[4]	x	x			x			x
	Ashley et al. (2014)[5]	x	x						x
QOL-BMT	Grant et al. (1992)[6]	x	x	x					
FACT-BMT	McQuellon et al. (1997)[7]	x	x			x		x	x
MDASI-CAR	Wang et al. (2023)[8]	x				x	x		
MY-POS	Osbourne et al. (2015)[9]	x	x			x			
	Ramsenthaler et al. (2017)[10]	x	x	x		x		x	x
	Davies et al. (2017)[11]		x	x		x			x
SBQ-LC	Hughes et al. (2022)[12]	x	x	x					
SCNS-SF 34	Boyes et al. (2009)[13]	x				x	x	x	x

**Measurement properties based on the COSMIN taxonomy[14]

Appendix 6: Instrument cards for the shortlisted PRO measures

Instrument Name	The Symptom Burden Questionnaire™
**Acronym or alternate names**	SBQ™
**Domains/Outcomes assessed**	Breathing, pain, circulation, fatigue, memory, thinking & communication, movement, sleep, ear, nose, and throat symptoms, stomach and digestion, muscles and joints, mental health and wellbeing, skin and hair, eyes, female reproductive and sexual health, male reproductive and sexual health, other symptoms, impact on daily life
**Plain language summary**	• Questionnaire to rate symptom severity, functional disability, additional symptoms, and overall health.<br>• 16 outcome domains each forming a standalone scales<br>• Completion time varies according to number of scales administered<br>• Approximate ePRO completion time for full measure (17 scales): 10 minutes
**Description**	To measure symptom burden (severity, frequency, interference) of long COVID
**Generic or condition specific**	Condition-specific (Post-Acute COVID-19 Syndrome, known as Long COVID)
**Age range**	Adults (18+ years)
**Number of subscales**	Multi-dimensional item bank with 17 standalone scales:<br>• 16 scales measuring symptoms:<br>- Breathing (7 items),<br>- Pain (4 items)<br>- Circulation (5 items)<br>- Fatigue (4 items)<br>- Memory, Thinking & Communication (10 items)<br>- Movement (3 items)<br>- Sleep (4 items)<br>- Ear, Nose, and Throat (14 items)<br>- Stomach and Digestion (8 items)<br>- Muscles and Joints (9 items)<br>- Mental Health and Wellbeing (9 items)<br>- Skin and Hair (8 items)<br>- Eyes (10 items)<br>- Female Reproductive and Sexual Health (7 items)<br>- Male Reproductive and Sexual Health (3 items)<br>- Other symptoms (18 items)<br>• 1 scale measuring symptom interference/Impact on Daily Life (8 items)
**Number of questions**	131 (123 items measuring symptoms and 8 items measuring symptom interference/impact on daily life)
**Link to online review copy of questionnaire**	qrco.de/bdzbL7
**Recall period**	In the last 7days
**Average time to complete**	Times vary depending on number of scales administered. Approximately 10 minutes to complete the full SBQ as an electronic PRO measure<br>15 to 20 minutes – paper and pen version
**Administer to**	Interviewer administered<br>Self-administered
**How to access a copy**	Visit the University of Birmingham website
**Licensing requirements**	A license is required for use. There is no charge for non-commercial/academic and/or clinical use. A fee applies for commercial licenses.
**Versions available (including translations)**	• Original instrument: English (UK)<br>• 6 translations – Arabic, Chinese, English (US), German, Indonesian, Japanese
**Scoring information**	• Dichotomous: Yes/No responses within some scales; 4-point Likert/Likert-type Scale ranging from 0: "None"/"Never"/"Not at all" to 3: "Severe"/"Always"/"Severely"<br>• Higher score = higher symptom burden
**Evidence of validation of other measurement properties**	Hughes SE, Haroon S, Subramanian A, McMullan C, Aiyegbusi OL, Turner GM, Jackson L, Davies EH, Frost C, McNamara G, Price G, Matthews K, Camaradou J, Ormerod J, Walker A, Calvert MJ. Development and validation of the symptom burden questionnaire for long covid (SBQ-LC): Rasch analysis. BMJ. 2022 Apr 27;377:e070230. doi: 10.1136/bmj-2022-070230.

Instrument Name	Quality of Life in Adult Cancer Survivors Scale
Acronym or alternate names	QLACS
Domains/Outcomes assessed	• Five cancer-specific domains were identified (appearance concerns, financial problems, distress over recurrence, family-related distress, and benefits of cancer).<br>• Seven generic quality of life domains (negative feelings, positive feelings, cognitive problems, sexual problems, physical pain, fatigue, and social avoidance).
Plain language summary	This PRO Measure assesses quality of life in long term cancer survivors. It has also been evaluated for use with short term cancer survivors.
Description	A quality-of-life measure designed to assess issues relevant to long term cancer survivors.
Generic or condition specific	Condition-specific (cancer patients)
Age range	Adults
Number of subscales	2
Number of questions	47
Link to online review copy of questionnaire	https://qrco.de/bedTUw
Recall period	In the past four weeks
Average time to complete	Not stated
Administer to	Not stated
How to access a copy	See Appendix A in Avis, N.E., Smith, K.W., McGraw, S. *et al.* Assessing Quality of Life in Adult Cancer Survivors (QLACS). *Qual Life Res* **14**, 1007–1023 (2005). https://doi.org/10.1007/s11136-004-2147-2
Licensing requirements	Free to use
Versions available (including translations)	Original instrument: English<br>Translations available
Scoring information	• Scores for each domain are the sum (after appropriate reverse scoring) of the individual item scores(1 = ‘‘Never’’ through 7 = ‘‘Always’’).<br>• Generic and Cancer-Specific summary scores are calculated by summing the relevant domains (excluding *benefits of cancer*, which is scored as a distinct domain only)<br>• The Generic and Cancer Specific Summary scales are scored so that higher scores represent more problems or lower QOL.
Evidence of validation of other measurement properties	Avis, N.E., Smith, K.W., McGraw, S. *et al.* Assessing Quality of Life in Adult Cancer Survivors (QLACS). *Qual Life Res* **14**, 1007–1023 (2005). https://doi.org/10.1007/s11136-004-2147-2<br><br>Ashley L, Smith AB, Jones H, Velikova G, Wright P. Traditional and Rasch psychometric analyses of the Quality of Life in Adult Cancer Survivors (QLACS) questionnaire in shorter-term cancer survivors 15 months post-diagnosis. Journal of Psychosomatic Research. 2014 Oct 1;77(4):322-9.

**Instrument Name**	**Functional Assessment of Cancer Therapy – Bone Marrow Transplant**
**Acronym or alternate names**	FACT-BMT
**Domains/Outcomes assessed**	FACT-G domains:<br>• Physical Well-being (PWB) (7 items)<br>• Social / Family Well-being (SWB) (7 items)<br>• Emotional Well-being (EWB) (6 items)<br>• Functional Well-being (FWB) (7 items)<br>+ Additional 23 items (Bone Marrow Transplantation subscale, BMTS)
**Plain language summary**	• The Functional Assessment of Cancer Therapy (FACT)-Bone Marrow Transplantation (BMT) is composed of the FACT-General (FACT-G) and a 23-item BMT-specific subscale (BMTS).<br>• 39 translations
**Description**	To assess quality of life in bone marrow transplant patients.
**Generic or condition specific**	Condition-specific (cancer patients undergoing bone marrow transplant)
**Age range**	Adults (18 to 64 years)
**Number of subscales**	FACT-G has 4 subscales (Physical well-being, social/family well-being, emotional well-being, functional well-being) + BMT subscale
**Number of questions**	50 (27 for the FACT-G + 23 for FACT-BMTS)
**Link to online review copy of questionnaire**	https://qrco.de/bedQJ8
**Recall period**	The past 7 days
**Average time to complete**	10 to 15 minutes
**Administer to**	Interviewer administered<br>Self-administered
**How to access a copy**	Consult FACIT.org website
**Licensing requirements**	License required. Use of the English version is free for anyone, although permission should be obtained for any situation. Translations carry a licensing fee of $1,500 per Latin language and $2,000 per non-Latin language for commercial, for-profit studies. Fees are typically waived for investigator-initiated research, student research and clinical use.
**Versions available (including translations)**	Original language: English<br>39 translations
**Scoring information**	• 5-point Likert/Likert-type scale from 0 = "not at all" to 4 = "very much")<br>• Global score (range 0 to 200)<br>• Scores by domains (PWB (range 0-28), SWB (range 0-28), EWB (range 0-24), FWB (range 0-28), FACT-G (range 0-108), BMTS (range 0-92), Trial Outcome Index (TOI=PWB+FWB+BMTS; range 0-148)<br>• Manual scoring template, some items are reverse scored. Subscale scores, total scores and TOI scores possible. SAS/SPSS algorithms available.
**Evidence of validation of other measurement properties**	McQuellon, R., Russell, G., Cella, D. *et al.* Quality of life measurement in bone marrow transplantation: development of the Functional Assessment of Cancer Therapy-Bone Marrow Transplant (FACT-BMT) scale. *Bone Marrow Transplant* 19, 357–368 (1997). https://doi.org/10.1038/sj.bmt.1700672
**Additional comments**	User manual available upon registration

Instrument Name	MD Anderson Symptom Inventory – CAR
Acronym or alternate names	MDASI-CAR
Domains/Outcomes assessed	CAR-T specific symptoms, and Interference
Plain language summary	To assess the severity of multiple symptoms and the impact of symptoms on daily functioning<br>Patients with symptoms caused by cancer and cancer treatment with focus on CAR-T cell therapies<br>Approximate completion time (paper version): 5 minutes<br>Translations available<br>Costs apply.
Description	A tool to assess symptom burden and daily functioning in patients after CAR-T cell therapy.
Generic or condition specific	Condition-specific (Cancer patients receiving CAR-T cell therapies)
Age range	Adults (18+ years)
Number of subscales	Single scale comprised of MDASI core symptom items and CAR-specific items
Number of questions	29
Link to online review copy of questionnaire	Contact Authors
Recall period	Past 24 hrs
Average time to complete	Approximately 5 min
Administer to	Self-completed
How to access a copy	qrco.de/bedR1o
Licensing requirements	Licensing fees and a $100 processing fee may apply. Fees vary depending on the type and extent of use, the setting, and who is sponsoring the research (either in full or in part). License fees apply for use in clinical practice and funded research studies.
Versions available (including translations)	Submit request via website or contact author
Scoring information	0-10 numeric rating scale where 0 = not present and 10 = as bad as you can imagine
Evidence of validation of other measurement properties	Whisenant MS, Srour SA, Williams LA, Subbiah I, Griffin D, Ponce D, Kebriaei P, Neelapu SS, Shpall E, Ahmed S, Wang XS. The Unique Symptom Burden of Patients Receiving CAR T-Cell Therapy. Semin Oncol Nurs. 2021 Dec;37(6):151216. doi:0.1016/j.soncn.2021.151216. https://doi.org/10.1016/j.soncn.2021.151216<br><br>Wang, Xin Shelley ; Srour, Samer A. ; Mendoza, Tito ; Whisenant, Meagan ; Subbiah, Ishwaria ; Gonzalez, Elizabeth ; Kamal, Mona ; Shen, Shu-En ; Cleeland, Charles ; Kebriaei, Partow ; Rezvani, Katayoun ; Neelapu, Sattva ; Ahmed, Sairah ; Shpall, Elizabeth<br>British journal of haematology, 2023, Vol.201 (4), p.738-746<br>https://doi.org/10.1111/bjh.18677
Additional comments	User guide can be found at: MD Anderson Symptom Inventory (MDASI) | MD Anderson Cancer Center

Instrument Name	Myeloma Patient Outcome Score
Acronym or alternate names	MyPOS
Domains/Outcomes assessed	Health-Related Quality of Life - HRQL (incl. health status)<br>3 Domains: Symptoms, functioning and emotional response, healthcare support
Plain language summary	MyPOS is a module of the Palliative Care Outcome Scale (POS), extended by myeloma-specific concerns.<br>Designed for use in clinical settings
Description	To measure quality of life in patients with multiple myeloma or follicular lymphoma
Generic or condition specific	Condition-specific (patients with multiple myeloma or follicular lymphoma)
Age range	Adult (18+ years)
Number of subscales	• Symptoms (13 items)<br>• Functioning and Emotional Response (17 items)<br>• Healthcare Support (3 items)<br>• + 3 additional items which are not scored
Number of questions	30
Link to online review copy of questionnaire	https://qrco.de/bedtzK
Recall period	Over the past week
Average time to complete	8 minutes
Administer to	Self-administered
How to access a copy	Contact Kings College London. E-mail: csipostranslations@kcl.ac.uk
Licensing requirements	Unknown, contact King College London. E-mail: csipostranslations@kcl.ac.uk
Versions available (including translations)	Original instrument: English (UK 2 translations)
Scoring information	5-point Likert/Likert-type scale + open-ended questions<br>("never" or "none" to "always" or "severe"<br>Higher score = Worse quality of life
Evidence of validation of other measurement properties	Osborne TR, Ramsenthaler C, de Wolf-Linder S, Schey SA, Siegert RJ, Edmonds PM, Higginson IJ. Understanding what matters most to people with multiple myeloma: a qualitative study of views on quality of life. BMC Cancer. 2014 Jul 9;14:496<br><br>Osborne TR, Ramsenthaler C, Schey SA, Siegert RJ, Edmonds PM, Higginson IJ. Improving the assessment of quality of life in the clinical care of myeloma patients: the development and validation of the Myeloma Patient Outcome Scale (MyPOS). BMC Cancer. 2015 Apr 14;15:280. doi: 10.1186/s12885-015-1261-6<br><br>Ramsenthaler C, Gao W, Siegert RJ, Schey SA, Edmonds PM, Higginson IJ. Longitudinal validity and reliability of the Myeloma Patient Outcome Scale (MyPOS) was established using traditional, generalizability and Rasch psychometric methods. Qual Life Res. 2017 Nov;26(11):2931-2947.<br><br>Davies JM, Osborne TR, Edmonds PM, Schey SA, Devereux S, Higginson IJ, Ramsenthaler C. The Myeloma Patient Outcome Scale is the first quality of life tool developed for clinical use and validated in patients with follicular lymphoma. Eur J Haematol. 2017 May;98(5):508-516.
Additional comments	

Hughes et al. Measure selection for an electronic patient-reported outcome (ePRO) system for CAR T-cell therapy patients: a modified Delphi consensus study

Instrument Name	Quality of Life Bone Marrow Transplant Survivors
**Acronym or alternate names**	QOL-BMT
**Domains/Outcomes assessed**	Physical well-being; Psychological well-being; Social concerns; Spiritual well-being.
**Plain language summary**	The instrument is based on conceptualization of quality of life which includes the four domains of physical well-being, psychological well-being, social concerns, and spiritual well-being.<br>Approximate completion time (paper version): 15 Min<br>No translations available<br>Cost: Free for use
**Description**	The instrument has two components. The first component consists of 20 forced-choice and open-ended items that relate to patient demographics and other patient characteristics. **The second component contains 64 QOL items using 10-point response scales.**
**Generic or condition specific**	Condition-specific (Bone Marrow transplant survivors)
**Age range**	Adults (18+ years)
**Number of subscales**	Four domains (subscales)
**Number of questions**	84 (64 related to domains, 22 additional patient information)
**Link to online review copy of questionnaire**	https://qrco.de/bedR7m
**Recall period**	At this time
**Average time to complete**	15 mins
**Administer to**	Self-administered
**How to access a copy**	Nursing Research and Education Resources | Beckman Research Institute | City of Hope
**Licensing requirements**	Free to use, require no further permission.
**Versions available (including translations)**	Original instrument: English (US)<br>No translations
**Scoring information**	10-point numerical score where all items are coded to reflect 0 = worst outcome/negative QOL to 10 = best outcome/positive QOL.<br>Some items are reversed-scored<br>Subscale and total scores may be calculated<br>Higher score = better quality of life
**Evidence of validation of other measurement properties**	Grant M, Ferrell B, Schmidt GM, Fonbuena P, Niland JC, & Forman SJ. Measurement of quality of life in bone marrow transplant survivors. Quality of Life Research, 1992; 1(6): 375-384 (PubMed abstract - https://doi.org/10.1007/bf00704432)
**Additional comments**	Instruction manual with the tool is available.

Instrument Name	Supportive Care Needs Survey – Short Form 34
**Acronym or alternate names**	SCNS-SF34
**Domains/Outcomes assessed**	The 34 items of SCNS-SF34 map to the following five domains of need: psychological, health system & information, physical & daily living, patient care & support and sexuality.
**Plain language summary**	• 5 domains<br>• Short form version of the SCNS<br>• Measures needs of adults with cancer.
**Description**	A self-administered questionnaire designed to measure the perceived needs of adults diagnosed with cancer.
**Generic or condition specific**	Condition-specific (Cancer patients)
**Age range**	18+ years
**Number of subscales**	5
**Number of questions**	34
**Link to review copy of questionnaire**	https://qrco.de/bedt91<br>See also Appendix 2 of the user manual
**Recall period**	In the last month
**Average time to complete**	15-20 mins
**Administer to**	Self-administered<br>Interview
**How to access a copy**	Contact Authors – CheRP@newcastle.edu.au
**Licensing requirements**	Contact authors, Supportive Care Needs Survey – Long Form 59 @ Centre for Health Research & Psycho-oncology, 2003, all right reserved
**Versions available (including translations)**	Original instrument: English<br>Translations – Chinese, Japanese, Spanish
**Scoring information**	It assesses whether issues of need have been experienced, which of the issues experienced remain unmet needs, and the magnitude of such needs, on a five-point response scale (1 = "no need, not applicable"; 2 = no need, satisfied; 3 = "low need"; 4 = "moderate need"; 5 = "high need").<br>A standardized Likert summated score with values ranging from 0 to 100 can be calculated for each domain, with a higher score reflecting a higher level of need
**Evidence of validation of other measurement properties**	Boyes, A., Girgis, A. and Lecathelinais, C. (2009), Brief assessment of adult cancer patients' perceived needs: development and validation of the 34-item Supportive Care Needs Survey (SCNS-SF34). Journal of Evaluation in Clinical Practice, 15: 602-606. https://doi.org/10.1111/j.1365-2753.2008.01057.x<br><br>Bonevski B, Sanson-Fisher R, Girgis A, Burton L, Cook P, Boyes A et al (the Supportive Care Review Group). Evaluation of an instrument to assess the needs of patients with cancer. Cancer 2000;88:226-37<br><br>McElduff P, Boyes A, Zucca A, Girgis A. Supportive care needs survey: a guide to administration, scoring and analysis. Newcastle: Centre for Health Research & Psycho-oncology. 2004 Jan.
**Additional comments**	Manual available (McElduff et al. 2004)

Hughes et al. Measure selection for an electronic patient-reported outcome (ePRO) system for CAR T-cell therapy patients: a modified Delphi consensus study

Appendix 7: Example screenshots from Delphi Round 1 Online Survey

Q23

How relevant is each questionnaire listed below to the measurement of the symptoms and side effects of CAR-T cell therapy? (Click here to see a description of the symptoms included in the PRO-CAR-T™ system. Click on the name of the questionnaire to see a description of the instrument, including a copy of the questionnaire). *

	0 - Not at all relevant	1	2	3	4	5 - Extremely relevant
Functional Assessment of Cancer Therapy - Bone Marrow Transplant (FACT-BMT)	○	○	○	○	○	○
MD Anderson Symptom Inventory - CAR (MDASI-CAR)	○	○	○	○	○	○
Quality of Life bone marrow Transplant Survivors (QoL-BMT)	○	○	○	○	○	○
The Symptom Burden Questionnaire (SBQ)	○	○	○	○	○	○

Comment:

Q24

To what extent does each questionnaire cover ALL of the symptoms included in the PRO-CAR-T™ system (Please click here for a list of symptoms). Click the name of the questionnaire to view a description of the instrument and view the review copy. *

	0 - Not at all comprehensive	1	2	3	4	5 - Extremely comprehensive
Functional Assessment of Cancer Therapy - Bone Marrow Transplant (FACT-BMT)	○	○	○	○	○	○
MD Anderson Symptom Inventory - CAR (MDASI-CAR)	○	○	○	○	○	○
Quality of Life bone marrow Transplant Survivors (QoL-BMT)	○	○	○	○	○	○
The Symptom Burden Questionnaire (SBQ)	○	○	○	○	○	○

Comment:

Q25

For each questionnaire, please rate how easy the questionnaire is to understand and complete. *

	0 - Very difficult	1 - Difficult	2 - Easy	3 - Very easy
Functional Assessment of Cancer Therapy - Bone Marrow Transplant (FACT-BMT)	○	○	○	○
MD Anderson Symptom Inventory - CAR (MDASI-CAR)	○	○	○	○
Quality of Life bone marrow Transplant Survivors (QoL-BMT)	○	○	○	○
The Symptom Burden Questionnaire (SBQ)	○	○	○	○

Comment:

Q26

How relevant is each questionnaire listed below to the measurement of the impacts of cancer and cancer treatment, specifically thinking about CAR-T cell therapy.
(Click here to see a description of the impacts measured by the PRO-CAR-T™ system. Click on the name of the questionnaire to see a description of the instrument, including a copy of the questionnaire). *

	0 - Not at all relevant	1	2	3	4	5 - Extremely relevant
Functional Assessment of Cancer Therapy - Bone Marrow Transplant (FACT-BMT)	○	○	○	○	○	○
Myeloma Patient Outcome Scale (MyPOS)	○	○	○	○	○	○
Quality of Life Bone Marrow Transplant survivors (QoL-BMT)	○	○	○	○	○	○
Quality of Life in Adult Cancer Survivors Scale (QLACS)	○	○	○	○	○	○
Supportive Care Needs Survey – Short Form 34 (SCNC-SF34)	○	○	○	○	○	○

Comment:

Q27

To what extent does each questionnaire cover ALL of the impacts of disease and treatment included in the PRO-CAR-T™ system (Please click here for a list of impacts). Click the name of the questionnaire to view a description of the instrument and view the review copy. *

	0 - Not at all comprehensive	1	2	3	4	5 - Extremely comprehensive
Functional Assessment of Cancer Therapy - Bone Marrow Transplant (FACT-BMT)	○	○	○	○	○	○
Myeloma Patient Outcome Scale (MyPOS)	○	○	○	○	○	○
Quality of Life Bone Marrow Transplant Survivors (QoL-BMT)	○	○	○	○	○	○
Quality of Life in Adult Cancer Survivors Scale (QLACS)	○	○	○	○	○	○
Supportive Care Needs Survey – Short Form 34 (SCNC-SF34)	○	○	○	○	○	○

Comment:

Q28

For each questionnaire, please rate how easy the questionnaire is to understand and complete. *

	0 - Very difficult	1 - Difficult	2 - Easy	3 - Very easy
Functional Assessment of Cancer Therapy - Bone Marrow Transplant (FACT-BMT)	○	○	○	○
Myeloma Patient Outcome Scale (MyPOS)	○	○	○	○
Quality of Life Bone Marrow Transplant Survivors (QoL-BMT)	○	○	○	○
Quality of Life in Adult Cancer Survivors Scale (QLACS)	○	○	○	○
Supportive Care Needs Survey – Short Form 34 (SCNC-SF34)	○	○	○	○

Comment:

Hughes et al. Measure selection for an electronic patient-reported outcome (ePRO) system for CAR T-cell therapy patients: a modified Delphi consensus study

Appendix 8: Results of Fisher's exact test showing exact probability values for each of the candidate PROMs for the indicators of relevance, comprehensiveness and ease of understanding per domain

(N.B.: values in bold denote a significant association between stakeholder group and indicator rating where p ≤ 0.05).

Domain: Symptom Burden

PRO Measure	Relevance	Comprehensiveness	Ease of Understanding
FACT-BMT	1·00	0·63	0·35
MDSAI-CAR	1·00	1·00	1·00
QoL-BMT	1·00	1·00	0·63
SBQ	0·53	0·53	1·00

Domain: Impacts of Disease and Treatment

PRO Measure	Relevance	Comprehensiveness	Ease of Understanding
FACT-BMT	0·30	0·57	1·00
MyPOS	0·60	0·63	0·15
QoL-BMT	0·57	1·00	0·60
QLACS	1·00	0·26	1·00
SCNC-SF34	1·00	1·00	1·00

Hughes et al. Measure selection for an electronic patient-reported outcome (ePRO) system for CAR T-cell therapy patients: a modified Delphi consensus study

References

1. Khatsuria F, McMullan C, Aiyegbusi OL, et al. Development of a conceptual framework for an electronic patient-reported outcome (ePRO) system measuring symptoms and impacts of CAR T-cell therapies in patients with haematological malignancies. Lancet Oncol. 2024;25(10):e476–e488.

2. Aiyegbusi OL, Kyte D, Cockwell P, et al. Measurement properties of patient-reported outcome measures (PROMs) used in adult patients with chronic kidney disease: a systematic review protocol. BMJ Open. 2016; 6(10):e012014.

3. Database of systematic reviews • COSMIN [Internet]. COSMIN. 2017 [cited 2024 Oct 24]. Available from: https://www.cosmin.nl/database/

4. Avis NE, Smith KW, McGraw S, Smith RG, Petronis VM, Carver CS. Assessing Quality of Life in Adult Cancer Survivors (QLACS). Quality of Life Research. 2005; 14(4):1007–23.

5. Ashley L, Smith AB, Jones H, Velikova G, Wright P. Traditional and Rasch psychometric analyses of the Quality of Life in Adult Cancer Survivors (QLACS) questionnaire in shorter-term cancer survivors 15months post-diagnosis. Journal of Psychosomatic Research. 2014; 77(4):322–9.

6. Grant M, Ferrell B, Schmidt GM, Fonbuena P, Niland JC, Forman SJ. Measurement of quality of life in bone marrow transplantation survivors. Quality of Life Research. 1992; 1(6):375–84.

7. McQuellon R, Russell G, Cella D, et al. Quality of life measurement in bone marrow transplantation: development of the Functional Assessment of Cancer Therapy-Bone Marrow Transplant (FACT-BMT) scale. Bone Marrow Transplantation. 1997; 19(4):357–68.

8. Wang XS, Srour SA, Mendoza T, et al. Development and validation of a patient-reported outcome measure to assess symptom burden after chimeric antigen receptor T-cell therapy. British journal of haematology. 2023; 201(4):738–46.

9. Osborne TB, Ramsenthaler C, Schey S, Siegert RJ, Edmonds P, Higginson IJ. Improving the assessment of quality of life in the clinical care of myeloma patients: the development and validation of the Myeloma Patient Outcome Scale (MyPOS). BMC Cancer. 2015; 15(1).

10. Ramsenthaler C, Gao W, Siegert RJ, Schey SA, Edmonds PM, Higginson IJ. Longitudinal validity and reliability of the Myeloma Patient Outcome Scale (MyPOS) was established using traditional, generalizability and Rasch psychometric methods. Quality of Life Research. 2017; 26(11):2931–47.

11. Davies JM, Osborne TR, Edmonds PM, et al. The Myeloma Patient Outcome Scale is the first quality of life tool developed for clinical use and validated in patients with follicular lymphoma. European journal of haematology. 2017; 98(5):508–16.

12. Hughes SE, Haroon S, Subramanian A, et al. Development and validation of the symptom burden questionnaire for long covid (SBQ-LC): Rasch analysis. BMJ. 2022; 377:e070230.

13. Boyes A, Girgis A, Lecathelinais C. Brief assessment of adult cancer patients' perceived needs: development and validation of the 34-item Supportive Care Needs Survey (SCNS-SF34). Journal of Evaluation in Clinical Practice. 2009; 15(4):602–6.

14. Mokkink LB, Terwee CB, Patrick DL, et al. The COSMIN checklist for assessing the methodological quality of studies on measurement properties of health status measurement instruments: an international Delphi study. Quality of life research : an international journal of quality of life aspects of treatment, care and rehabilitation. 2010; 19(4):539–49.

15. Jünger S, Payne SA, Brine J, Radbruch L, Brearley SG. Guidance on Conducting and REporting DElphi Studies (CREDES) in palliative care: Recommendations based on a methodological systematic review. Palliat Med. 2017; 31(8):684–706.
